# Genome sequence of an Australian kangaroo, *Macropus eugenii*, provides insight into the evolution of mammalian reproduction and development

**DOI:** 10.1186/gb-2011-12-8-r81

**Published:** 2011-08-19

**Authors:** Marilyn B Renfree, Anthony T Papenfuss, Janine E Deakin, James Lindsay, Thomas Heider, Katherine Belov, Willem Rens, Paul D Waters, Elizabeth A Pharo, Geoff Shaw, Emily SW Wong, Christophe M Lefèvre, Kevin R Nicholas, Yoko Kuroki, Matthew J Wakefield, Kyall R Zenger, Chenwei Wang, Malcolm Ferguson-Smith, Frank W Nicholas, Danielle Hickford, Hongshi Yu, Kirsty R Short, Hannah V Siddle, Stephen R Frankenberg, Keng Yih Chew, Brandon R Menzies, Jessica M Stringer, Shunsuke Suzuki, Timothy A Hore, Margaret L Delbridge, Amir Mohammadi, Nanette Y Schneider, Yanqiu Hu, William O'Hara, Shafagh Al Nadaf, Chen Wu, Zhi-Ping Feng, Benjamin G Cocks, Jianghui Wang, Paul Flicek, Stephen MJ Searle, Susan Fairley, Kathryn Beal, Javier Herrero, Dawn M Carone, Yutaka Suzuki, Sumio Sugano, Atsushi Toyoda, Yoshiyuki Sakaki, Shinji Kondo, Yuichiro Nishida, Shoji Tatsumoto, Ion Mandiou, Arthur Hsu, Kaighin A McColl, Benjamin Lansdell, George Weinstock, Elizabeth Kuczek, Annette McGrath, Peter Wilson, Artem Men, Mehlika Hazar-Rethinam, Allison Hall, John Davis, David Wood, Sarah Williams, Yogi Sundaravadanam, Donna M Muzny, Shalini N Jhangiani, Lora R Lewis, Margaret B Morgan, Geoffrey O Okwuonu, San Juana Ruiz, Jireh Santibanez, Lynne Nazareth, Andrew Cree, Gerald Fowler, Christie L Kovar, Huyen H Dinh, Vandita Joshi, Chyn Jing, Fremiet Lara, Rebecca Thornton, Lei Chen, Jixin Deng, Yue Liu, Joshua Y Shen, Xing-Zhi Song, Janette Edson, Carmen Troon, Daniel Thomas, Amber Stephens, Lankesha Yapa, Tanya Levchenko, Richard A Gibbs, Desmond W Cooper, Terence P Speed, Asao Fujiyama, Jennifer A M Graves, Rachel J O'Neill, Andrew J Pask, Susan M Forrest, Kim C Worley

**Affiliations:** 1 The Australian Research Council Centre of Excellence in Kangaroo Genomics, Australia; 2 Department of Zoology, The University of Melbourne, Melbourne, Victoria 3010, Australia; 3 Bioinformatics Division, The Walter and Eliza Hall Institute of Medical Research, Parkville, Victoria 3052, Australia; 4 Department of Mathematics and Statistics, The University of Melbourne, Melbourne, Victoria 3010, Australia; 5 Research School of Biology, The Australian National University, Canberra, ACT 0200, Australia; 6 Department of Molecular and Cell Biology, Center for Applied Genetics and Technology, University of Connecticut, Storrs, CT 06269, USA; 7 Faculty of Veterinary Science, University of Sydney, Sydney, NSW 2006, Australia; 8 Department of Veterinary Medicine, University of Cambridge, Madingley Rd, Cambridge, CB3 0ES, UK; 9 Institute for Technology Research and Innovation, Deakin University, Geelong, Victoria, 3214, Australia; 10 RIKEN Institute, 1-7-22 Suehiro-cho, Tsurumi-ku, Yokohama, Kanagawa 230-0045, Japan; 11 School of Marine and Tropical Biology, James Cook University, Townsville, Queensland 4811, Australia; 12 Department of Microbiology and Immunology, The University of Melbourne, Melbourne, Victoria 3010, Australia; 13 Leibniz Institute for Zoo and Wildlife Research, Alfred-Kowalke-Str. 17, Berlin 10315, Germany; 14 Laboratory of Developmental Genetics and Imprinting, The Babraham Institute, Cambridge, CB22 3AT, UK; 15 Department of Molecular Genetics, German Institute of Human Nutrition, Potsdam-Rehbruecke, Arthur-Scheunert-Allee 114-116, 14558 Nuthetal, Germany; 16 Department of Medical Biology, The University of Melbourne, Melbourne, Victoria 3010, Australia; 17 Biosciences Research Division, Department of Primary Industries, Victoria, 1 Park Drive, Bundoora 3083, Australia; 18 European Bioinformatics Institute, Wellcome Trust Genome Campus, Hinxton, Cambridge, CB10 1SD, UK; 19 Wellcome Trust Sanger Institute, Wellcome Trust Genome Campus, Hinxton, Cambridge, CB10 1SD, UK; 20 Department of Cell Biology, University of Massachusetts Medical School, Worcester, MA 01655, USA; 21 Graduate School of Frontier Sciences, The University of Tokyo, Chiba 277-8560, Japan; 22 National Institute of Genetics, Mishima, Shizuoka 411-8540, Japan; 23 Department of Computer Science and Engineering, University of Connecticut, Storrs, CT 06269, USA; 24 Human Genome Sequencing Center, Department of Molecular and Human Genetics Baylor College of Medicine, Houston, TX 77030, USA; 25 Australian Genome Research Facility, Melbourne, Victoria, 3052 and the University of Queensland, St Lucia, Queensland 4072, Australia; 26 Westmead Institute for Cancer Research, University of Sydney, Westmead, New South Wales 2145, Australia; 27 National Institute of Informatics, 2-1-2 Hitotsubashi, Chiyoda-ku, Tokyo 101-8430, Japan; 28 Department of Biological, Earth and Environmental Sciences, The University of New South Wales, Sydney, NSW 2052, Australia

## Abstract

**Background:**

We present the genome sequence of the tammar wallaby, *Macropus eugenii*, which is a member of the kangaroo family and the first representative of the iconic hopping mammals that symbolize Australia to be sequenced. The tammar has many unusual biological characteristics, including the longest period of embryonic diapause of any mammal, extremely synchronized seasonal breeding and prolonged and sophisticated lactation within a well-defined pouch. Like other marsupials, it gives birth to highly altricial young, and has a small number of very large chromosomes, making it a valuable model for genomics, reproduction and development.

**Results:**

The genome has been sequenced to 2 × coverage using Sanger sequencing, enhanced with additional next generation sequencing and the integration of extensive physical and linkage maps to build the genome assembly. We also sequenced the tammar transcriptome across many tissues and developmental time points. Our analyses of these data shed light on mammalian reproduction, development and genome evolution: there is innovation in reproductive and lactational genes, rapid evolution of germ cell genes, and incomplete, locus-specific X inactivation. We also observe novel retrotransposons and a highly rearranged major histocompatibility complex, with many class I genes located outside the complex. Novel microRNAs in the tammar HOX clusters uncover new potential mammalian HOX regulatory elements.

**Conclusions:**

Analyses of these resources enhance our understanding of marsupial gene evolution, identify marsupial-specific conserved non-coding elements and critical genes across a range of biological systems, including reproduction, development and immunity, and provide new insight into marsupial and mammalian biology and genome evolution.

## Background

The tammar wallaby holds a unique place in the natural history of Australia, for it was the first Australian marsupial discovered, and the first in which its special mode of reproduction was noted: '*their manner of procreation is exceeding strange and highly worth observing; below the belly the female carries a pouch into which you may put your hand; inside the pouch are her nipples, and we have found that the young ones grow up in this pouch with the nipples in their mouths. We have seen some young ones lying there, which were only the size of a bean, though at the same time perfectly proportioned so that it seems certain that they grow there out of the nipples of the mammae from which they draw their food, until they are grown up*' [1]. These observations were made by Francisco Pelseart, Captain of the ill-fated and mutinous Dutch East Indies ship Batavia in 1629, whilst shipwrecked on the Abrolhos Islands off the coast of Geraldton in Western Australia. It is therefore appropriate that the tammar should be the first Australian marsupial subject to an in-depth genome analysis.

**Figure 1:**
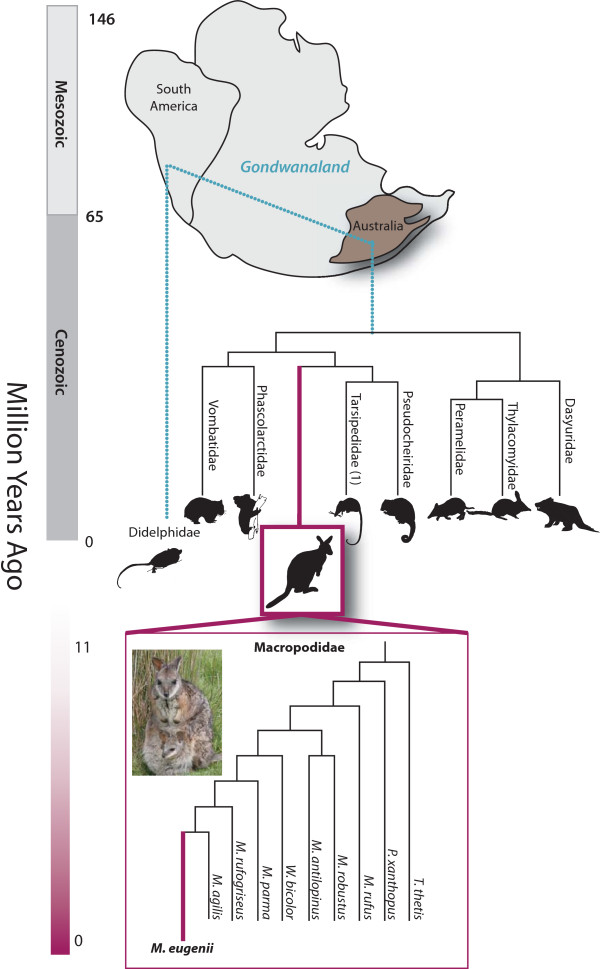
**Phylogeny of the marsupials**. Phylogenetic relationships of the orders of Marsupialia. Top: the placement of the contemporary continents of South America and Australia within Gondwanaland and the split of the American and Australian marsupials. Relative divergence in millions of years shown to the left in the context of geological periods. The relationship of the Macropodide within the Australian marsupial phylogeny shown is in purple with estimated divergence dates in millions of years [5,162,163]. Representative species from each clade are illustrated. Inset: phylogeny of the genus *Macropus *within the Macropodidae showing the placement of the model species *M. eugenii *(purple) based on [59]. Outgroup species are *Thylogale thetis *and *Petrogale xanthopus*.

Marsupials are distantly related to eutherian mammals, having shared a common ancestor between 130 and 148 million years ago [2-4]. The tammar wallaby *Macropus eugenii *is a small member of the kangaroo family, the Macropodidae, within the genus *Macropus*, which comprises 14 species [5] (Figure 1). The macropodids are the most specialized of all marsupials. Mature females weigh about 5 to 6 kg, and males up to 9 kg. The tammar is highly abundant in its habitat on Kangaroo Island in South Australia, and is also found on the Abrolhos Islands, Garden Island and the Recherche Archipelago, all in Western Australia, as well as a few small areas in the south-west corner of the continental mainland. These populations have been separated for at least 40,000 years. Its size, availability and ease of handling have made it the most intensively studied model marsupial for a wide variety of genetic, developmental, reproductive, physiological, biochemical, neurobiological and ecological studies [6-13].

In the wild, female Kangaroo Island tammars have a highly synchronized breeding cycle and deliver a single young on or about 22 January (one gestation period after the longest day in the Southern hemisphere, 21 to 22 December) that remains in the pouch for 9 to 10 months. The mother mates within a few hours after birth but development of the resulting embryo is delayed during an 11 month period of suspended animation (embryonic diapause). Initially diapause is maintained by a lactation-mediated inhibition, and in the second half of the year by photoperiod-mediated inhibition that is removed as day length decreases [14]. The anatomy, physiology, embryology, endocrinology and genetics of the tammar have been described in detail throughout development [6,11-13,15].

The marsupial mode of reproduction exemplified by the tammar with a short gestation and a long lactation does not imply inferiority, nor does it represent a transitory evolutionary stage, as was originally thought. It is a successful and adaptable lifestyle. The maternal investment is minimal during the relatively brief pregnancy and in early lactation, allowing the mother to respond to altered environmental conditions [11,12,15]. The tammar, like all marsupials, has a fully functional placenta that makes hormones to modulate pregnancy and parturition, control the growth of the young, and provide signals for the maternal recognition of pregnancy [14,16-18]. The tammar embryo develops for only 26 days after diapause, and is born when only 16 to 17 mm long and weighing about 440 mg at a developmental stage roughly equivalent to a 40-day human or 15-day mouse embryo. The kidney bean-sized newborn has well-developed forelimbs that allow it to climb up to the mother's pouch, where it attaches to one of four available teats. It has functional, though not fully developed, olfactory, respiratory, circulatory and digestive systems, but it is born with an embryonic kidney and undifferentiated immune, thermoregulatory and reproductive systems, all of which become functionally differentiated during the lengthy pouch life. Most major structures and organs, including the hindlimbs, eyes, gonads and a significant portion of the brain, differentiate while the young is in the pouch and are therefore readily available for study [11,12,19-24]. They also have a sophisticated lactational physiology with a milk composition that changes throughout pouch life, ensuring that nutrient supply is perfectly matched for each stage of development [25]. Adjacent teats in a pouch can deliver milk of differing composition appropriate for a pouch young and a young-at-foot [26].

Kangaroo chromosomes excited some of the earliest comparative cytological studies of mammals. Like other kangaroos, the tammar has a low diploid number (2n = 16) and very large chromosomes that are easily distinguished by size and morphology. The low diploid number of marsupials makes it easy to study mitosis, cell cycles [27], DNA replication [28], radiation sensitivity [29], genome stability [30], chromosome elimination [31,32] and chromosome evolution [33,34]. Marsupial sex chromosomes are particularly informative. The X and Y chromosomes are small; the basic X chromosome constitutes only 3% of the haploid genome (compared with 5% in eutherians) and the Y is tiny. Comparative studies show that the marsupial X and Y are representative of the ancestral mammalian X and Y chromosomes [35]. However, in the kangaroos, a large heterochromatic nucleolus organizer region became fused to the X and Y. Chromosome painting confirms the extreme conservation of kangaroo chromosomes [36] and their close relationship with karyotypes of more distantly related marsupials [37-40] so that genome studies are likely to be highly transferable across marsupial species.

The tammar is a member of the Australian marsupial clade and, as a macropodid marsupial, is maximally divergent from the only other sequenced model marsupial, the didelphid Brazilian grey short-tailed opossum, *Monodelphis domestica *[41]. The South American and Australasian marsupials followed independent evolutionary pathways after the separation of Gondwana into the new continents of South America and Australia about 80 million years ago and after the divergence of tammar and opossum (Figure 1) [2,4]. The Australasian marsupials have many unique specializations. Detailed knowledge of the biology of the tammar has informed our interpretation of its genome and highlighted many novel aspects of marsupial evolution.

## Sequencing and assembly (Meug_1)

The genome of a female tammar of Kangaroo Island, South Australia origin was sequenced using the whole-genome shotgun (WGS) approach and Sanger sequencing. DNA isolated from the lung tissue of a single tammar was used to generate WGS libraries with inserts of 2 to 6 kb (Tables S1 and S2 in Additional file 1). Sanger DNA sequencing was performed at the Baylor College of Medicine Human Genome Sequencing Center (BCM-HGSC), and the Australian Genome Research Facility using ABI3730xl sequencers (Applied BioSystems, Foster City, CA, USA). Approximately 10 million Sanger WGS reads, representing about 2 × sequence coverage, were submitted to the NCBI trace archives (NCBI BioProject PRJNA12586; NCBI Taxonomy ID 9315). An additional 5.9 × sequence coverage was generated on an ABI SOLiD sequencer at BCM-HGSC. These 25-bp paired-end data with average mate-pair distance of 1.4 kb (Table S3 in Additional file 1) [SRA:SRX011374] were used to correct contigs and perform super-scaffolding. The initial tammar genome assembly (Meug_1.0) was constructed using only the low coverage Sanger sequences. This was then improved with additional scaffolding using sequences generated with the ABI SOLiD (Meug_1.1; Table 1; Tables S4 to S7 in Additional file 1). The Meug_1.1 assembly had a contig N50 of 2.6 kb and a scaffold N50 of 41.8 kb [GenBank:GL044074-GL172636].

**Table 1:** Comparison of Meug genome assemblies

	Assembly version		
	
	1.0	1.1	2.0
Contigs (million)	1.211	1.174	1.111
N50 (kb)	2.5	2.6	2.91
Bases (Mb)	2546	2,536	2,574
Scaffolds	616,418	277,711	379,858
Max scaffold size	NA	472,108	324,751
Gaps (Mb)	NA	539	619
N50 (kb)	NA	41.8	34.3
Complex scaffolds	NA	128,563	124,674
Singleton scaffolds	NA	149,148	255,184
Co-linear with BACs	NA	87.2% (418)	93.4% (298)
Co-linear with ESTs	NA	82.3% (704)	86.7% (454)

Summary statistics for the tammar genome assemblies. These statistics indicate the extension and merging of contigs done to improve the assembly. The larger number of scaffolds and smaller scaffold N50 is a consequence of higher stringency in the 2.0 scaffolding workflow. The higher stringency isolated many contigs. However, the number of complex (that is, useful) scaffolds is similar between the assemblies. For co-linear estimates, the scaffolds were linearized and BACs and cDNA libraries were mapped against them. The 1.1 and 2.0 assemblies were validated against 169 BAC contigs and 84,718 ESTs (that were not incorporated into either genome assembly). We determined the percentage of contigs where the scaffolding matched the order and orientation when compared to BACs or ESTs (co-linear with BACs/ESTs). Parentheses indicate the total number of contigs identified after alignment to BAC contigs or ESTs.

The completeness of the assembly was assessed by comparison to the available cDNA data. Using 758,062 454 FLX cDNA sequences [SRA:SRX019249, SRA:SRX019250], 76% are found to some extent in the assembly and 30% are found with more than 80% of their length represented (Table S6 in Additional file 1). Compared to 14,878 Sanger-sequenced ESTs [GenBank:EX195538-EX203564, GenBank:EX203644-EX210452], more than 85% are found in the assembly with at least one half their length aligned (Table S7 in Additional file 1).

## Additional sequencing and assembly improvement (Meug_2)

### Contig improvement

The tammar genome assembly was further improved using additional data consisting of 0.3 × coverage by paired and unpaired 454 GS-FLX Titanium reads [SRA:SRX080604, SRA:SRX085177] and 5 × coverage by paired Illumina GAIIx reads [SRA:SRX085178, SRA:SRX081248] (Table S8 in Additional file 1). A local reassembly strategy mapped the additional 454 and Illumina data against Meug_1.1 contigs. Added data were used to improve the accuracy of base calls and to extend and merge contigs. The Meug_2.0 assembly [GenBank:ABQO000000000] (see also 'Data availability' section) has 1.111 million contigs with an N50 of 2.9 kb. Contigs were validated directly by PCR on ten randomly selected contigs. The assembly was also assessed by aligning 84,718 ESTs and 169 BAC sequences to the genome. The amount of sequence aligning correctly to the genome assembly showed modest improvement between Meug_1.1 and Meug_2.0 (Table 1; Table S9 in Additional file 1).

### Scaffolding and anchoring using the virtual map

Scaffolds were constructed using the previously mentioned Illumina paired-end libraries with insert sizes of 3.1 kb (8,301,018 reads) and 7.1 kb (12,203,204 reads), 454 paired-end library with an insert size of 6 kb and SOLiD mate pair library. The mean insertion distances for each library were empirically determined using paired reads where both ends mapped within the same contig and only those within three standard deviations from the mean were used for scaffolding. The contigs were ordered and oriented using Bambus [42], through three iterations of scaffolding to maximize the accuracy of the assembly. The highest priority was given to the library with the smallest standard deviation in the paired end distances, and the remaining libraries arranged in descending order. Initial scaffolding by Bambus was performed using five links as a threshold [43]. Overlapping contigs were identified and set aside before reiteration. This step was performed twice and the overlapping contigs pooled. The non-overlapping and overlapping contigs were then scaffolded independently. Any scaffolds found to still contain overlap were split apart. The resulting assembly has 324,751 scaffolds with an N50 of 34,279 bp (Table 1). Scaffolds were assigned to chromosomes by aligning them to markers from the virtual map [44], represented using sequences obtained from the opossum and human genomes [45]. We assigned 6,979 non-overlapping scaffolds (163 Mb or 6% of the genome assembly) to the seven autosomes. The vast majority of the genome sequence remained unmapped.

## Tammar genome size

The tammar genome size was estimated using three independent methods: direct assessment by quantitative PCR [46]; bivariate flow karyotyping and standard flow cytometry; and genome analyses based in the Sanger WGS reads, using the Atlas-Genometer [47]. These three approaches produced quite different genome size estimates (Tables S11 to S13 in Additional file 1) so the average size estimate, 2.9 Gb, was used for the purposes of constructing the Meug_2.0 integrated genome assembly. The smaller genome size of tammar compared to human is unlikely to be due to fewer genes or changes in gene size (Figure S1 in Additional file 2), but may be accounted for by the greatly reduced centromere size of 450 kb/chromosome and number (*n *= 8) [48] compared to the human centromere size of 4 to 10 Mb/chromosome (*n *= 23).

## Physical and linkage mapping

Novel strategies were developed for the construction of physical and linkage maps covering the entire genome. The physical map consists of 520 loci mapped by fluorescence *in situ *hybridization (FISH) and was constructed by mapping the ends of gene blocks conserved between human and opossum, thereby allowing the location of genes within these conserved blocks to be extrapolated from the opossum genome onto tammar chromosomes [37] (JE Deakin, ML Delbridge, E Koina, N Harley, DA McMillan, AE Alsop, C Wang, VS Patel, and JAM Graves, unpublished results). Three different approaches were used to generate a linkage map consisting of 148 loci spanning 1,402.4 cM or 82.6% of the genome [49]. These approaches made the most of the available tammar sequence (genome, BACs or BAC ends) to identify markers to increase coverage in specific regions of the genome. Many of these markers were also physically mapped, providing anchors for the creation of an integrated map comprising all 553 distinct loci included in the physical and/or linkage maps. Interpolation of segments of conserved synteny (mainly from the opossum assembly) into the integrated map then made it possible to predict the genomic content and organization of the tammar genome through the construction of a virtual genome map comprising 14,336 markers [44].

**Figure 2:**
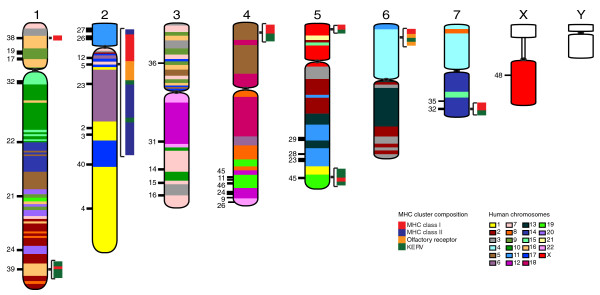
**Homology of tammar regions to the human karyotype, and location of major histocompatibility complex, classical class I genes and olfactory receptor gene**. Colored blocks represent the syntenic blocks with human chromosomes as shown in the key. A map of the locations of the tammar major histocompatibility complex (MHC) is shown on the right-hand side of each chromosome. The rearranged MHCs are on chromosome 2 and clusters of MHC class I genes (red) near the telomeric regions of chromosomes 1, 4, 5, 6, and 7. MHC class II genes are shown in blue, olfactory receptors are shown in orange and Kangaroo endogenous retroviral elements found within these clusters are shown in green. The location of the conserved mammalian OR gene clusters in the tammar genome are shown on the left-hand side of each chromosome. OR genes are found on every chromosome, except for chromosome 6 but including the X. The location of the OR gene clusters (numbers) are shown, and their approximate size is represented by lines of different thickness.

Mapping data were used to construct tammar-human (Figure 2) and tammar-opossum comparative maps in order to study genome evolution. Regions of the genome were identified that have undergone extensive rearrangement when comparisons between tammar and opossum are made. These are in addition to previously known rearrangements based on chromosome-specific paints [50]. For example, tammar chromosome 3, consisting of genes that are on nine human chromosomes (3, 5, 7, 9, 10, 12, 16, 17, 22; Figure 2) and the X have an extensive reshuffling of the gene order. Rearrangements on the remaining chromosomes are mostly the result of large-scale inversions. This enabled us to predict the ancestral marsupial karyotype, revealing that inversions and micro-inversions have played a major role in shaping the genomes of marsupials (JE Deakin, ML Delbridge, E Koina, N Harley, DA McMillan, AE Alsop, C Wang, VS Patel, and JAM Graves, unpublished results).

## Genome annotation

The Ensembl genebuild (release 63) for the Meug_1.0 assembly identified 18,258 genes by projection from high quality reference genomes. Of these, 15,290 are protein coding, 1,496 are predicted pseudo-genes, 525 are microRNA (miRNA) genes, and 42 are long non-coding RNA genes, though these are composed of just 7 different families: 7SK, human accelerated region 1F, CPEB3 ribozyme, ncRNA repressor of NFAT, nuclear RNase P, RNase MRP and Y RNA.

Since the coverage is low, many genes may be fragmented in the assembly or even unsequenced. The Ensembl genebuild pipeline scaffolds fragmented genes using comparative data and constructs 'GeneScaffolds'. There are 10,257 GeneScaffolds containing 13,037 genes. The annotation also contains 9,454 genes interrupted by Ns. To partially ameliorate the problems of missing genes, a number of BACs from targeted locations have been sequenced and annotated, including the HOX gene clusters (H Yu, Z-P Feng, RJ O'Neill, Y Hu, AJ Pask, D Carone, J Lindsay, G Shaw, AT Papenfuss, and MB Renfree, unpublished results), major histocompatibility complex (MHC) [51], X chromosome (ML Delbridge, B Landsdell, MT Ross, TP Speed, AT Papenfuss, JAM Graves, unpublished results), pluripotency genes, germ cell genes, spermatogenesis genes [52,53] and X chromosome genes. Findings from these are summarized in later sections of this paper.

### Expansion of gene families

Many genes evolve and acquire novel function through duplication and divergence. We identified genes that have undergone expansions in the marsupial lineage but remain largely unduplicated in eutherians and reptiles (Table S15 in Additional file 1). Both the tammar and opossum have undergone expansion of MHC class II genes, critical in the immune recognition of extracellular pathogens, and *TAP *genes that are responsible for loading endogenously derived antigens onto MHC class I proteins. Three marsupial-specific class II gene families exist: DA, DB and DC. Class II genes have undergone further duplications in the tammar and form two genomic clusters, adjacent to the antigen-processing genes [51]. The opossum has one *TAP1 *and two *TAP2 *genes, while the tammar has expanded *TAP1 *(two genes) and *TAP2 *(three genes) genes [51]. We also detected marsupial expansions linked to apoptosis (*NET1*, *CASP3*, *TMBIM6*) and sensory perception (olfactory receptors).

## Genomic landscape

### Sequence conservation

We next explored sequence conservation between tammar and opossum using sequence similarity as a sensitive model of conservation. We found that 38% of nucleotides in the tammar genome (Meug_1.0) could be aligned to the high-quality opossum genome (7.3×). Of the aligned sequence, 72% was unannotated, reflecting a high proportion of conserved non-coding regions between the marsupial species. The level of conservation between opossum and tammar varied from 36.0 to 40.9% across the different opossum chromosomes (Table S16 in Additional file 1). This variation seems modest and may be largely stochastic, but it is interesting to examine further. Opossum chromosome 1 has 40.6% sequence conservation with the tammar. The gene order between tammar and opossum chromosome 1 is also highly conserved. This may mean that within the tammar genome assembly scaffolds, the alignment is well anchored by conserved protein-coding genes, making the intergenic sequence easier to align. Thus this 'high' conservation may be largely due to inherent biases in the approach. Opossum chromosome X has the most conserved sequence compared to tammar (40.9%), despite the high level of rearrangement between the tammar and opossum X. Intriguingly, the proportion of conserved sequence on opossum chromosome X that is located in unannotated regions is also the highest of any chromosome (28.2%; Table S16 in Additional file 1) despite the level of rearrangement. This may indicate a significant number of non-coding regulatory elements on the X chromosome. The mechanism of X inactivation in marsupials is not well understood. Examination of transcription within individual nuclei shows that there is at least regional coordinated expression of genes on the partially inactive X [54-56]. It would be interesting to determine whether these conserved non-coding sequences are involved.

### GC content

The average GC content based upon the assembly Meug_2.0 is 38.8% (Table 2), while the GC content based upon cytometry is 34%. This is lower than the GC content for human (41%) but similar to opossum (38%). The tammar X also has a GC content (34%) lower than that of the opossum X (42%). Thus, tammar chromosomes are relatively GC poor. The proportion of CpGs in the tammar genome is higher than that of the opossum, but similar to human (Table 2). The GC content was also calculated from RIKEN full-length cDNA pools and varied from 44% to 49% across tissue types (Table S17 in Additional file 1), indicating that the lower GC content of the tammar genome is contained within non-exonic regions.

**Table 2:** Comparison of repeat landscape in tammar and other mammals

	Tammar	Opossum	Platypus	Human	Mouse
Total assembly size (Gb)	2.7	3.48	2.3	2.88	2.55
Interspersed repeats (%)					
Total	52.8	52.2	44.6	45.5	40.9
LINE/non-LTR retroelements	28.6	29.2	21.0	20.0	19.6
SINE	11.7	10.4	22.4	12.6	7.2
ERV	3.9	10.6	0.47	8.1	9.8
DNA transposon	2.9	1.7	1.1	2.8	0.8
C+G (%)	38.8	37.7	45.5	40.9	41.8
CpG (%)	3.5	2.3	NA	3.7	3.9

Comparative analyses of the interspersed repeat content in the tammar and other sequenced mammalian genomes. Repeat modeller combined dataset includes *ab initio *annotation of *de novo *repeats. ERV, endogenous retroviral element; LTR, long terminal repeat; NA, not available.

### Repeats

The repeat content of the tammar wallaby genome was assessed using RepeatMasker, RepeatModeler and *ab initio *repeat prediction programs. The Repbase database of consensus repeat sequences was used to identify repeats in the genome derived from known classes of elements [57] (Table 2). RepeatModeler uses a variety of *ab initio *tools to identify repetitive sequences irrespective of known classes [58]. After identification, the putative *de novo *repeats were mapped against the Repbase repeat annotations using BLAST. Any *de novo *repeat with at least 50% identity and coverage was annotated as that specific Repbase element. All putative *de novo *repeats that could not be annotated were considered *bona fide*, *de novo *repeats. The results from the database and *de novo *RepeatMasker annotations were combined, and any overlapping annotations were merged if they were of the same class of repeat element. Overlapping repeats from different classes were reported; therefore, each position in the genome may have more than one unique annotation.

The total proportion of repetitive sequence in the tammar was found to be 52.8%, although this is probably an underestimate resulting from the low coverage. This is similar to the repeat content of the opossum genome (52.2%). The proportion of LINEs and SINEs was also similar between opossum and tammar; however, the overall content for long terminal repeat (LTR) elements was significantly below that observed for any other mammal (only 3.91%) with the exception of the platypus (about 0.47%). Interestingly, 36 elements were identified that were tammar-specific, including novel LTR elements (25), SINEs (1), LINEs (4) and DNA elements (3). Moreover, analyses of the small RNA pools that emanate from repeats (see below) allowed for identification of a novel SINE class that is rRNA derived and shared among all mammals (J Lindsay, DM Carone, E Murchison, G Hannon, AJ Pask, MB Renfree, and RJ O'Neill, unpublished results; MS Longo, LE Hall, S Trusiak, MJ O'Neill, and RJ O'Neill, unpublished results).

Given the unique small size of the tammar centromere, estimated to cover only 450 kb [48], the genome was further scanned for putative pericentric regions using our previously annotated centromere repeat elements [59]. We identified 66,256 contigs in 53,241 scaffolds as having centromeric sequences and these were further examined for repeat structure. Analyses of these regions confirms the proposed punctate distribution of repeats within pericentromeric regions of the tammar [48,60] and indicate the absence of monomeric satellite repeats in the centromeres of this species (J Lindsay, S Al Seesi, RJ O'Neill, unpublished results) compared with many others (reviewed in [61,62]).

## The tammar transcriptome

Sequencing of the tammar genome has been augmented by extensive transcriptomic sequencing from multiple tissues using both Sanger sequencing and the Roche 454 platform by a number of different groups. Transcriptome datasets collected are summarized in Table S17 in Additional file 1 and are described in more detail in several companion papers. Sequences from the multiple tissues have been combined to assess the assembly and annotation, and to provide a resource that supplements the low coverage tammar genome by identifying and adding unsequenced and unannotated genes.

**Figure 3:**
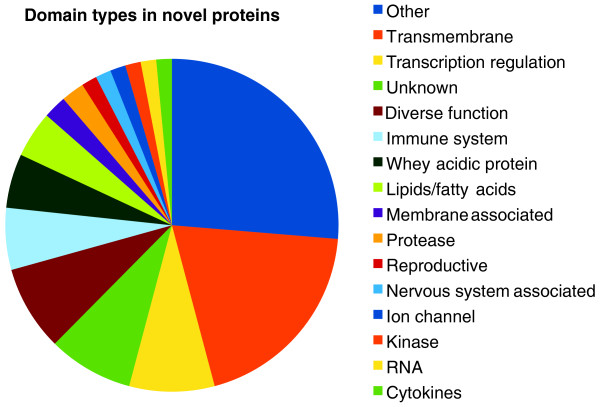
**Classification of novel tammar genes**. Summary of protein domains contained within translated novel ESTs isolated from the tammar transcriptomes. A large proportion of unique genes contain receptor or transcriptional regulator domains. The next largest classes of unique ESTs were immune genes, whey acidic protein and lipid domain containing genes. These findings suggest a rapid diversification of genes associated with immune function and lactation in the tammar.

Transcriptomes of the testis [DDBJ:FY644883-FY736474], ovary [DDBJ:FY602565-FY644882], mammary gland [GenBank:EX195538-EX203564, GenBank:EX203644-EX210452], gravid uterus [DDBJ:FY469875-FY560833], hypothalamus [DDBJ:FY560834-FY602565) and cervical and thoracic thymus [SRA:SRX019249, SRA:SRX019250] were sequenced. Each dataset was aligned to the assembly (Meug_1.0) using BLASTN. The proportion of reads that mapped varied between approximately 50% and 90% depending on the tissues of origin (Figure S2a Additional file 3). Of the successfully mapped reads, the proportion aligning to annotated genes (Ensembl annotation or 2 kb up- or downstream) were more similar between libraries (Figure S2b in Additional file 3). However, the lowest rates at which reads mapped to annotated genes in the genome were observed in transcripts from the two thymuses and the mammary gland. The former is unsurprising as a large number of immune genes are expressed in the thymus and are likely to be more difficult to annotate by projection due to their rapid evolution. The lower rate at which these ESTs aligned to annotated genes in mammary gland may reflect the highly sophisticated and complex lactation of marsupials (reviewed in [12]), a conclusion supported by the large number of unique genes identified with whey acidic protein and lipid domains (Figure 3). The mammary transcriptome may also contain a large number of immune transcripts. Together, these findings suggest a high degree of innovation in immune and lactation genes in the tammar. Previous analyses revealed that about 10% of transcripts in the mammary transcriptome were marsupial-specific and up to 15% are therian-specific [63]. Conversely, the high proportion of reads mapping to annotated genes in the testis and ovary (> 80%) suggest that there is significant conservation of active genes involved in reproduction between mammalian species (see section on 'Reproductive genes'

The testis, ovary, hypothalamus and gravid uterus full-length cDNA libraries were end-sequenced at RIKEN to evaluate composition and complexity of each transcriptome. We produced 360,350 Sanger reads in total (Table S18a in Additional file 1). Reads were clustered and the ratio of the clusters to reads was used as an estimate of the tissue's transcriptomic complexity. The hypothalamus showed the highest complexity (44.3%), whereas ovary showed the lowest (18.8%). We then looked for representative genes in each library by aligning reads to the Refseq database using BLASTN. For example, homologues of *KLH10 *and *ODF1/2*, both of which function in spermatogenesis and male fertility, were found to be highly represented in the testis library (4.3% and 3.5% respectively). The hypothalamus library was rich in tubulin family genes (7.9% of reads), and hormone-related genes such as *SST *(somatostatin; 1.8% of reads) (see Table S18b in Additional file 1 for details).

### Highly divergent or tammar-specific transcripts

Based upon stringent alignments to Kyoto Encyclopedia of Genes and Genomes genes (E-value < 10^-30^), it was initially estimated that up to 17% of ovary clusters, 22% of testis clusters, 29% of gravid uterus clusters and 52% of hypothalamus clusters were tammar-specific or highly divergent. Unique genes were identified by clustering of the EST libraries (to remove redundancy) followed by alignment of the unique reads to dbEST (NCBI) with BLASTN [64] using an E-value threshold of 10^-5^. We identified 4,678 unique ESTs (6.1%) from a total of 76,171 input ESTs (following clustering) and used these for further analyses. Sequences were translated using OrfPredictor [65] and passed through PfamA [66] for classification. Of the unique genes that could be classified using this approach, many appear to be receptors or transcriptional regulators (Figure 3). A large number of unique ESTs contained whey acidic protein and lipid domains, common in milk proteins, suggesting a rapid diversification of these genes in the tammar genome. An EST containing a unique zona pellucida domain was also identified. Detailed expression was examined for 32 unique genes isolated from the RIKEN testis RNA-Seq pool. Of the initial 32, 11 were gonad-specific. Spatial expression of five of these genes was examined by *in situ *hybridization in adult testes and ovaries. One gene was germ cell-specific, two genes had weak signals in the somatic tissue and the remaining two genes were not detected.

### Small RNAs

**Figure 4:**
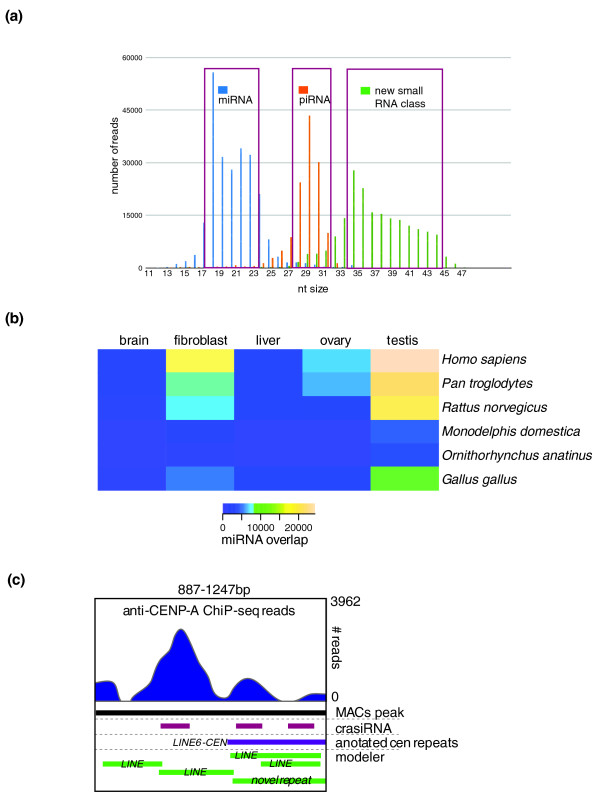
**A survey of both conserved and novel small RNAs in the tammar genome**. **(a) **Size ranges of the major classes of small RNAs. The x-axis shows number of reads mapped to the tammar genome while the size of the read in nucleotides is on the y-axis. Boxes denote each major class analyzed in the tammar. Classes targeted for sequencing and full annotation include the miRNAs (18 to 22 nucleotides), the piRNAs (28 to 32 nucleotides) and the newly discovered crasiRNAs (35 to 45 nucleotides). **(b) **Five tammar miRNA libraries (brain, liver, fibroblast, ovary and testis) were pooled and mapped to the tammar genome. miRNAs with a complete overlap with miRBase entries mapped to the tammar genome were considered conserved and annotated according to species. Heat map showing the frequency of conserved mirBase entries per tissue and per species as identified in the tammar. A high degree of overlap (that is, conservation) was observed between tammar and human for fibroblast and testis, but a relatively low degree of overlap was observed for the brain. **(c) **The complex tammar centromere. Genome browser view of chromatin immunoprecipitation-sequencing (ChIP-Seq) for DNA bound by the centromere-specific histone CENP-A mapped to a centromeric contig (top, blue). Nucleotide position on the contig is shown on the x-axis and depth of reads shown on the y-axis. Tracks illustrated: MACs peak (model-based analyses of Chip-Seq (black); locations for mapped reads of crasiRNAs (red); location of annotated centromere sequences (in this example, the centromeric LINE L6; purple); modeler repeat prediction track (green). crasiRNAs co-localize to DNA found in CENP-A-containing nucleosomes and are enriched in regions containing known centromere sequences.

Recently, it has become clear that small RNAs are essential regulatory molecules involved in a variety of pathways, including gene regulation, chromatin dynamics and genome defense. While many small RNA classes appear to be well conserved, such as the miRNAs, it has become evident that small RNA classes can also evolve rapidly and contribute to species incompatibilities [67-70]. Our analyses of the tammar small RNAs focused on known classes of small RNAs, miRNAs, and Piwi-interacting RNAs (piRNAs), as well as a novel class first identified in the tammar wallaby, centromere repeat-associated short interacting RNAs (crasiRNAs) [48] (Figure 4a).

Small RNAs in the size range 18 to 25 nucleotides, including miRNAs, from neonatal fibroblasts, liver, ovary, testis and brain were sequenced [GEO:GSE30370, SRA:SRP007394] and annotated. Following the mapping pipeline (Supplementary methods in Additional file 1), hairpin predictions for the precursor sequence within the tammar genome for each small RNA in this class were used. Those small RNAs derived from a genomic location with a *bona fide *hairpin were classified as miRNA genes and further analyzed for both conserved and novel miRNAs. Of those annotated in Ensembl, one was confirmed as a novel tammar miRNA gene and a further 56 as putative miRNA genes. Using a cross-database mapping scheme targeting both miRBase [71-74] and the tammar genome assembly (Supplementary methods in Additional file 1), 11% of miRNAs in the tammar tissues analyzed were related to previously annotated miRNAs (Figure 4b). However, the majority of miRNA alignments in the genome did not overlap with previously identified miRNAs and are thus considered novel. Combining these datasets with the gene annotations, 147 target genes were conserved with other mammals. Of these, four were shared between mouse and tammar and twelve were shared between human and tammar, thus indicating that the tammar miRNA repository might provide new targets for study in these species. Moreover, there were nine novel target genes in the tammar genome, pointing to both tammar-specific miRNA regulation as well as potentially novel targets in human that were previously unknown. Small RNAs were also identified in the HOX clusters (see 'HOX gene patterning in the limb' section below).

piRNAs are predominantly found in ovaries and testes [69,75,76]. Global comparisons to RepBase and our *de novo *repeat database show that the overall composition of tammar piRNAs in testis is similar in terms of repeat element type (that is, SINEs, LINEs, and so on) to that observed for other species. In addition, there were ovary-specific piRNAs derived from *de novo *tammar repeats, which may contribute to the observed hybrid incompatibility observed in this group of marsupial mammals [60,77-79].

The first identification of crasiRNAs (35 to 42 nucleotides) found that they contain centromere repeat-derived sequences specific to the retroelement KERV (kangaroo endogenous retrovirus) [48,60]. Approximately 68% of repeat-associated crasiRNAs mapped within viral-derived repeats (such as KERV) [80], SINE, and LINE elements (J Lindsay, S Al Seesi, RJ O'Neill, unpublished results). Many of these elements mapped to centromeres using primed *in situ *labeling (PRINS), and mapped to scaffolds enriched for centromere-specific repeats and CENP-A-containing nucleosomes (as determined by ChIP-seq) [GEO:GSE30371, SRA:SRP007562], confirming that this pool consists of centromeric elements (Figure 4c). Closer examination of this sequence pool and the progenitor sequences within the genome uncovered a distinct motif specific to the crasiRNAs, which may indicate novel biogenesis (J Lindsay, S Al Seesi, and RJ O'Neill, unpublished results).

## Immunity

The organization of the tammar MHC is vastly different from that of other mammals [81,82]. Rather than forming a single cluster, MHC genes are found on every chromosome, except the sex chromosomes (Figure 2). The MHC itself is found on chromosome 2q and contains 132 genes spanning 4 Mb [51]. This region was sequenced using a BAC-based Sanger sequencing strategy as it did not assemble well from the low-coverage sequencing. An expansion of MHC class II genes is accompanied by duplication of antigen-processing genes. The seven classical MHC class I genes are all found outside the core MHC region. KERVs may have contributed to this re-organization (Figure 2).

The tammar wallaby has two thymuses: a thoracic thymus (typically found in all mammals) and a dominant cervical thymus. Based on digital gene expression profiles both thymuses appear functionally equivalent and drive T-cell development [83]. Transcriptomic sequencing also shows that both thymuses express genes that mediate distinct phases of T-cell differentiation, including the initial commitment of blood stem cells to the T lineage (for example, *IL-7R*, *NOTCH1*, *GATA3*, *SPI1*, *IKZF1*), the generation of T-cell receptor diversity and development of the thymic environment (for example, *TRAF6*, *TP63 *and *LTBR*). In the thymus transcriptomes, we identified and annotated 34 cytokines and their receptors (10 chemokines, 22 interleukins and 2 interferons), 22 natural killer cell receptors (20 leukocyte receptor complex (LRC) genes and 2 natural killer complex (NKC) genes), 3 antimicrobial peptides (2 beta-defensins and 1 cathelicidin), post-switch immunoglobulin isotypes *IgA *and *IgG *and *CD4 *and *CD8 *T-cell markers.

At birth, the altricial pouch young is exposed to a variety of different bacterial species in the pouch. These include *Acinetobacter *spp., *Escherichia coli *and *Corynebacteria *spp. [84]. These bacteria remain in the pouch despite the female tammar extensively cleaning the pouch by licking before birth. To survive in this pathogen-laden environment, the immunologically naive neonate is reliant on immune factors, which are transmitted from the mother through the milk. The sequencing of the genome uncovered a family of cathelicidin genes, which are expressed in the mammary gland during lactation and encode powerful antimicrobial peptides. These peptides may provide unique opportunities to develop novel therapeutics against emerging multidrug-resistant superbugs.

Due to the rapid evolution of immune genes, a high proportion of tammar immune genes were not annotated using automated annotation pipelines. For this reason an Immunome Database for Marsupials and Monotremes has been established [85]. This database contains over 5,000 marsupial and monotreme immune sequences from a variety of EST projects, as well as expert-curated gene predictions. Marsupial chemokine, interleukin, natural killer cell receptor, surface receptor and antimicrobial peptide gene sequences are also available. Genomic evidence confirms that the marsupial immune system is on par with the eutherian immune system in terms of complexity.

## Sex chromosomes

**Figure 5:**
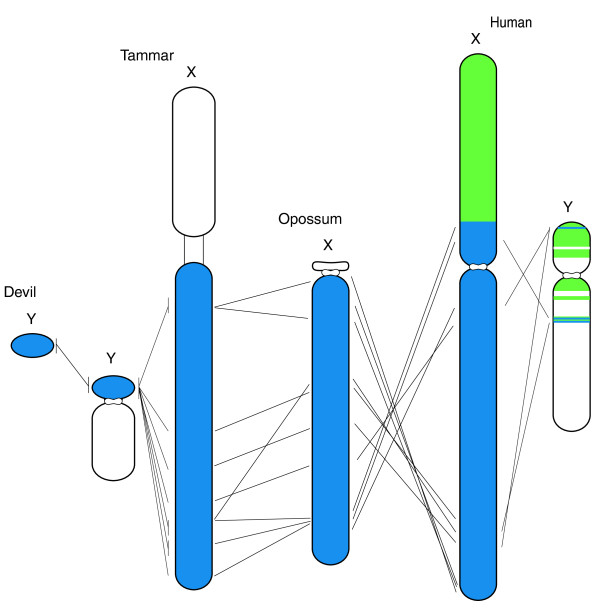
**Comparative map of X and Y chromosomes**. Comparison of X/Y shared gene locations on the tammar wallaby, grey short-tailed opossum and human X chromosomes. Blue represents the X conserved region, which is common to all therian X chromosomes. Green represents the X added region, which is on the X in eutherian mammals, but autosomal in marsupial mammals. Ten genes have been identified on the short arm of the tammar Y chromosome, all with a partner on the X, and an orthologue on the Tasmanian devil Y. In contrast, only four genes on the human Y have a partner on the conserved region of the X.

Marsupial sex chromosomes have been shown to represent the ancestral sex chromosomes, to which an autosomal region was fused early in the eutherian radiation. Thus, the basic marsupial X shares homology with the long arm and pericentric region of the human X [35,36]. The tammar Y shares only five genes with the degraded eutherian Y [86] (Figure 5).

Marsupial sex chromosomes lack the autosomal addition and so are expected to be smaller than those of eutherian mammals. The opossum X is about 97 Mb (Table S12 in Additional file 1). The larger size of the tammar X (150 Mb) reflects the addition of a heterochromatic arm containing satellite repeats and the nucleolus-organizing region [59]. Of the 451 protein coding genes on the opossum X chromosome, 302 have orthologues in the tammar Ensembl gene build. Gene mapping indicates that the gene order within the tammar X is scrambled with respect to both the opossum and human X chromosomes [37]. This scrambling of the marsupial X contrasts to the eutherian X chromosome, which is almost identical in gene content and order between even the most distantly related taxa [87,88]. The rigid conservation of the eutherian X was hypothesized to be the result of strong purifying selection against rearrangements that might interrupt a chromosome-wide mechanism to effect X-chromosome inactivation. Consistent with this hypothesis, inactivation on the scrambled marsupial X is incomplete, locus-specific, and does not appear to be controlled by an inactivation center [54,56].

In many marsupial species the Y chromosome is a minute element of about 12 Mb. The tammar Y is larger, as the result of the addition to the X and Y in the early macropodid radiation of a heterochromatic long arm that contained the nucleolar organizing region (NOR) and NOR-associated repeats [59]. Degradation of the Y removed active rDNA genes but left repetitive sequences with homology to the NOR-bearing short arm of the X [89,90]. The tammar Y chromosome bears at least ten genes, which are all located on the tiny short arm of the Y (reviewed in [91]) (V Murtagh, N Sankovic, ML Delbridge, Y Kuroki, JJ Boore, A Toyoda, KS Jordan, AJ Pask, MB Renfree, A Fujiyama, JAM Graves and PD Waters, unpublished results). All ten have orthologues on the Y of a distantly related Australian dasyurid marsupial, the Tasmanian devil, implying that the marsupial Y chromosome is conserved (Figure 5). It has degraded more slowly than the eutherian Y, which retains only four (human) or five (other mammals) genes from the ancient XY pair [91,92].

Like most genes on the human Y, all of these tammar Y genes have an X partner, from which they clearly diverged. Some tammar Y genes are expressed exclusively in the testis (for example, the marsupial-specific *ATRY *[93]), but most have widespread expression. Phylogenetic analysis of the X and Y copies of these ten tammar XY genes indicate that marsupial Y genes have a complex evolutionary history.

## X chromosome inactivation

Epigenetic silencing of one X chromosome occurs in female mammals as a means of dosage compensation between XX females and XY males. Classic work on kangaroos established that X inactivation occurs in marsupials, but is paternal, incomplete and tissue-specific [94] and apparently occurs in the absence of the *XIST *controlling element [95,96]. Using tammar sequence to isolate X-borne genes and study their expression at the level of individual nuclei using RNA *in situ *hybridization, it has been found that different genes have a characteristic frequency of expression from one or both loci, suggesting that it is the probability of expression rather than the rate of transcription that is controlled [54]. The absence of clustering of high- or low-expressing genes has not so far provided evidence for an inactivation center. It appears that X inactivation in marsupials, like eutherians, uses a repressive histone-mediated gene silencing, and although inactive marks are not identical [55,56], they do have H3K27 trimethylation and targeting to the perinucleolar compartment [97].

## Reproductive genes

Marsupials differ from eutherian mammals primarily in their unique mode of reproduction. In contrast to mice and humans, in which sexual differentiation occurs *in utero*, the altricial 440 mg tammar neonate has indifferent gonads on the day of birth and does not undergo gonadal sex determination until approximately 2 days later (testis) and 8 days later (ovary) [22]. This postnatal differentiation of the gonads therefore provides an unparalleled model for studying sex determination and sexual differentiation and enables experimental manipulation not possible in eutherian species. We have shown that almost all genes critical for testis and ovarian development are highly conserved between the tammar, mouse and human at the molecular level [98,99], but their precise role in gonadogenesis may differ between the mammalian groups.

### Gonadal differentiation genes

*ATRX *is an ultra-conserved, X-linked gene essential for normal testis development in humans. Marsupials are unique among the mammals in that they have orthologues of this gene on both their X and Y chromosomes (*ATRX *and *ATRY*, respectively). Almost all X-linked genes once shared a partner on the Y, but the vast majority of these have been lost during its progressive degeneration. The Y-linked *ATRX *orthologue was lost in the eutherian lineage before their radiation, but was retained in the marsupial lineage. *ATRY *shows functional specialization, and is exclusively expressed in the developing and adult testis of the tammar, while tammar *ATRX *is broadly expressed, but is absent in the developing testis, unlike eutherians [93]. The distribution of *ATRX *mRNA and protein in the developing gonads is ultra-conserved between the tammar and the mouse [100], and is found within the germ cells and somatic cells. *ATRX *therefore appears to have a critical and conserved role in normal development of the testis and ovary that has remained unchanged for up to 148 million years of mammalian evolution [100].

*Desert hedgehog *(*DHH*) is another essential signaling molecule required for normal testicular patterning in mice and humans. Members of the hedgehog family of secreted proteins act as intercellular transducers that control tissue patterning across the entire embryo. Like other hedgehog proteins, DHH signals through the PTCH receptors 1 and 2 [101]. *DHH*, *PTCH1 *and *PTCH2 *in the tammar are highly conserved with their eutherian orthologues. However, unlike in eutherian mammals, *DHH *expression is not restricted to the testes during tammar development, but is also detected in the developing ovary (WA O'Hara, WJ Azar, RR Behringer, MB Renfree, and AJ Pask, unpublished results). Furthermore, hedgehog-signaling inhibitors disrupt both testicular and ovarian differentiation [101]. Together, these data confirm a highly conserved role for DHH in the formation of both the male and female tammar gonad.

**Figure 6:**
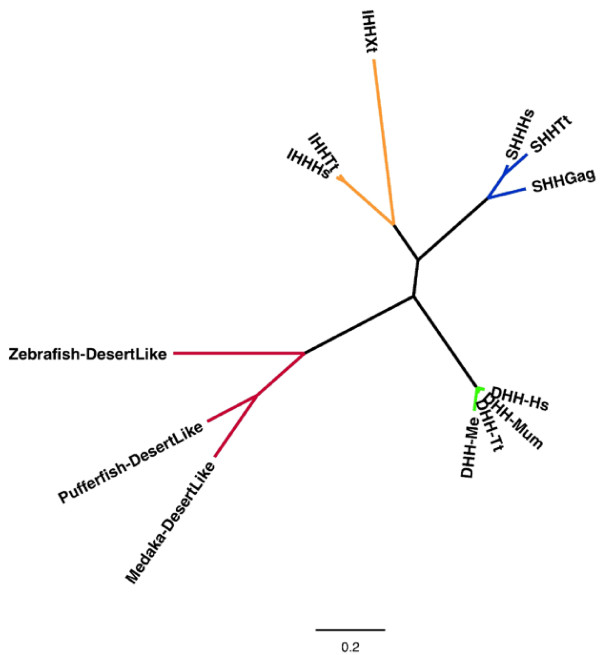
***Desert hedgehog *phylogeny**. A phylogenetic tree showing the relationship of the SHH, IHH, DHH, and fish desert-like genes. Each group is composed of representatives from mammalian and non-mammalian species. The mammalian DHH group (green) clusters tightly and forms a separate linage to the fish DHH-like genes (red), which are no more closely related to DHH than they are to vertebrate IHH (yellow) and SHH (blue). Hs, human; Tt, dolphin; Xt, *Xenopus*; Gag, chicken; Mum, mouse; Me, tammar.

Most interestingly, *DHH *is clearly a mammal-specific gonadal development gene. Hedgehog orthologues that are described as DHH in non-mammalian vertebrates actually form a distinct lineage no more closely related to mammalian DHH than they are to Sonic hedgehog (SHH) or Indian hedgehog (IHH) orthologues (Figure 6). Thus, *DHH *is the only mammal-specific gonadal development gene other than *SRY *so far discovered. In the tammar *PTCH2 *a novel exon (exon 21a) was detected that is not annotated in any eutherian PTCH2 proteins (WA O'Hara, WJ Azar, RR Behringer, MB Renfree, and AJ Pask, unpublished results). These analyses suggest that DHH evolved recently in vertebrates, yet acquired a critical role in mammalian gonadal development before the eutherian-marsupial divergence. However, the role of DHH in gonadogenesis has become more specialized to the testis in the eutherian lineage.

### Germ cell genes

The differentiation of the somatic cell lineages in the ovary and testis, mediated by the pathways described above, is critical for the subsequent development of the germ cells. Germ cells carry the genetic information from one generation to the next, making them arguably the most important cell lineage in the body. Comparative analyses of the genes essential for mouse and human germ cell development using the tammar genome presented an unexpected paradox. It was presumed that the genes mediating germ cell specification and development in mammals would be highly conserved because this cell lineage is critical for species' survival. However, our analyses indicate that many genes are rapidly evolving and likely to be controlled by specific elements in each mammalian lineage.

Orthologues of genes critical for the specification and development of eutherian germ cells, including *BMP4*, *PRDM1 *and *PRDM14*, were identified in the tammar genome. The tammar genome also contains transcripts for *DDX4 *(*VASA*) [102]. One transcript encodes a full length protein and the other has exon 4 spliced out. *In silico *analysis and 3' RACE showed that tammar *DDX4 *also utilizes more than one polyA signal [102]. The significance of these differentially spliced and alternatively polyadenylated *DDX4 *transcripts is unknown but may represent alternative mechanisms for controlling *DDX4 *expression; the 3' untranslated region of *DDX4 *in many species controls the localization, stabilization and translation of the gene [103]. Some genes expressed in murine primordial germ cells (PGCs) but not essential for their development lack marsupial orthologues. *Stella *is expressed in PGCs and in pluripotent cells but mice lacking *Stella *do not have any defects in germ cell specification or development [104]. In humans, *STELLA *is located on chromosome 12p13, a region known for structural chromosomal changes that are commonly associated with germ cell tumor formation. This region contains a cluster of genes, including *NANOG *and *GDF3 *[105], that are expressed in pluripotent cells. The syntenic region in the tammar and opossum contains *NANOG *and *GDF3 *but *STELLA *is absent, suggesting it evolved only recently in the eutherian lineage. Similarly, interferon inducible transmembrane protein (Ifitm)3 is produced in cells competent to form PGCs in mice [106], and both Ifitm3 and Ifitm1 are thought to mediate migration of PGCs from the posterior mesoderm into the endoderm [107]. Ifitm proteins 1 and 3 are expressed in early murine PGCs [106,108] but deletion of the locus containing Ifitm1 and Ifitm3 has no apparent effect on germ cell specification or migration [109]. The tammar genome contains several IFITM orthologues, some expressed in the early embryo, as in the mouse. The low sequence conservation between marsupial and eutherian IFITM orthologues suggests that the IFITMs may not be critical for mammalian germ cell development.

### Spermatogenesis genes

The genes regulating the later differentiation of the germ cells into mature oocytes and spermatocytes, especially those controlling spermatogenesis, are much more conserved between marsupials and eutherians than the signals that trigger their initial development. In eutherian mammals, there are a disproportionately high number of genes involved in spermatogenesis located on the X chromosome [110]. From the genome analyses in the tammar, it is clear that some of these genes were originally autosomal, and others appear to be on the ancestral X of the therian ancestor.

*AKAP4*, a scaffold protein essential for fibrous sheath assembly during spermatogenesis, is X-linked in the tammar as it is in eutherian mammals and maintains a highly conserved role in spermatogenesis [111]. In contrast, the *Kallman syndrome gene 1 *(*KAL1*) is X-linked in eutherians but autosomal in the tammar, located on chromosome 5p in a block of genes transposed to the X chromosome in an ancestral eutherian [52]. Despite its different chromosomal location, *KAL1 *is highly conserved and expressed in neuronal tissues as well as in the developing and adult gonads throughout spermatogenesis. Thus *KAL1 *probably evolved its role in mammalian gametogenesis before its relocation to the eutherian X [52]. Another eutherian X-linked gene, *TGIFLX *is absent from the tammar genome, but its progenitor, *TGIF2*, is present and appears to function in gametogenesis. Once again, this suggests that the gene had a role in spermatogenesis before its retrotransposition to the eutherian X [53]. These genomic and functional analyses not only shed light on the control of mammalian spermatogenesis, but also on genome evolution. These data support the theory that the X chromosome has selectively recruited and maintained spermatogenesis genes during eutherian evolution.

## Developmental genes

The segregation of the first cell lineages and specification of embryonic and extra-embryonic cell lineages have been studied extensively in the mouse. However, the mouse has a highly specialized embryogenesis, quite different from that of other mammals. Unlike a typical eutherian blastocyst with its inner cell mass, the tammar conceptus forms a unilaminar blastocyst of approximately 100 cells that lacks a readily defined pluriblast in the form of an inner cell mass. It can undergo a prolonged period of diapause. Thus, these differences highlight the developmental plasticity of mammalian embryos and genome analysis may provide comparative data that clarify the underlying control mechanisms of early mammalian development.

### Pluripotency genes

The tammar embryo develops when the embryonic disc forms on the blastocyst surface. The difference in embryo specification raises many interesting questions about early marsupial and mammalian development in general. After the differentiation of the embryonic area, the tammar embryo proper develops in a planar fashion on the surface of the embryonic vesicle. This makes the study of early embryonic events and morphogenesis easier to observe and manipulate than in the complicated egg cylinder formed in the mouse.

It is still unknown how the cells are specified in the unilaminar blastocyst that will go on to form the embryo in the tammar, but in the polyovular dasyurid marsupials, and also in the opossum, there appears to be cellular polarity in cleavage stages (reviewed in [112]). Whether the signals that regulate specification and induction are the same or different from those that regulate the specification of the eutherian mammal inner cell mass is under investigation. However, *POU5F1 *expression is limited to pluripotent cell types in the tammar as in eutherians. Marsupials additionally have a *POU2 *orthologue that is similarly expressed in pluripotent tissues but is also expressed in a broad range of adult tissues, suggesting that unlike *POU5F1*, the role of POU2 may function in maintaining multipotency in adult stem cells [113]. In the tammar, opossum and platypus genomes, but not in eutherian genomes, *POU2 *is an ancient vertebrate paralogue of *POU5F1 *[113,114]. Tammar wallaby *POU2 *is co-expressed in embryonic pluripotent tissues with *POU5F1 *but is also expressed in a broad range of adult tissues, suggesting it may also additionally function in maintaining multipotency in adult marsupial stem cells [113].

Orthologues of the vast majority of early developmental genes characterized in the mouse were identified in the tammar genome, including those encoding key transcription factors, such as *POU5F1*, *SOX2*, *NANOG*, *CDX2*, *EOMES*, *GATA4*, *GATA6 *and *BRACHYURY*. Genes encoding components of key signaling pathways in early development are largely conserved between tammar and mouse. One exception is *TDGF1 *(also called *CRIPTO*), which is present in eutherians but absent from the genome in tammar (as well as in those of opossum, platypus and non-mammalian vertebrates). *TDGF1 *encodes a co-receptor of NODAL signaling, which has a central role in early germ layer formation and axial specification in the mouse and in self-renewal of human embryonic stem cells [115]. Thus, *TDGF1 *is eutherian-specific, while the related paralogue *CFC1 *(also called *CRYPTIC*) is widely conserved in all vertebrates. This suggests the evolution of partly divergent roles for NODAL signaling in early embryonic patterning among mammals.

### Embryonic patterning

**Figure 7:**
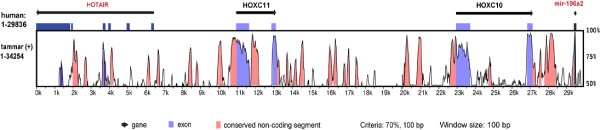
***HOX *genes in the tammar**. mVISTA comparison of partial HOXC cluster highlights conserved HOX genes and non-coding RNAs between human and tammar. In the coding regions, *HOXC11 *and *HOXC10 *are highly conserved between human and tammar. In the intergenic regions, some conserved regions shown are non-coding RNAs (long non-coding RNA such as *HOTAIR*, and miRNAs such as mir-196) or unknown motifs participating in gene expression and regulation. The percentage of identities (50 to 100%) (Vertical axis) is displayed in the coordinates of the genomic sequence (horizontal axis).

Once the early embryo is formed, the body plan must be established. The HOX genes are essential regulators of embryonic patterning in all animals, mediating the specification of structures along the anterior-posterior axis. In the tammar, as in all vertebrates, the HOX genes are arranged in four clusters. The clusters are low in repetitive elements compared to the rest of the genome (H Yu, Z-P Feng, RJ O'Neill, Y Hu, AJ Pask, D Carone, J Lindsay, G Shaw, AT Papenfuss, and MB Renfree, unpublished results). The tammar HOX clusters have a high degree of both conservation and innovation in the protein-coding and non-coding functional elements relative to eutherian mammals (Figure 7). Intronic regions are mostly divergent, but have isolated regions of high similarity corresponding to important enhancer elements. In eutherians, the clusters contain conserved intronic non-coding RNAs that are likely to participate in gene regulation [116]. Using the tammar genome, a new tetrapod miRNA was identified by conservation analysis and confirmed by RT-PCR to be expressed in fibroblasts (H Yu, Z-P Feng, RJ O'Neill, Y Hu, AJ Pask, D Carone, J Lindsay, G Shaw, AT Papenfuss, and MB Renfree, unpublished results). In addition, two novel miRNAs were characterized that are not conserved in eutherian mammals (Figure 7).

The HOX clusters also contain a number of genes that are transcribed into long non-coding RNAs [117,118]. Three long non-coding RNAs previously identified in the mouse were identified in the tammar HOX gene clusters. *HOX *antisense intergenic RNA myeloid 1 (*HOTAIRM1*), located between *HOXA1 *and *HOXA2*, is conserved in mammals and shows myeloid-specific expression [119]. Similarly, *HOXA11 *antisense (*HOXA11AS*), located between *HOXA13 *and *HOXA11*, is only conserved in mammals and is expressed during the human menstrual cycle [120]. Interestingly, antisense intergenic RNA (*HOTAIR*), located between *HOXC12 *and *HOXC11*, was conserved between human, mouse and tammar only in exons 3 and 6 (Figure 7). *HOTAIR *is an important trans-regulator that controls *HOXD *but not *HOXC *gene expression during limb development [116,121] and participates in reprogramming chromatin state to promote cancer metastasis [122]. The expression of *HOTAIR *was confirmed by RT-PCR in the tammar, suggesting an important and conserved regulatory role for this gene. The functional consequences of the marsupial-specific miRNAs and variation in the long non-coding RNAs are yet to be determined, but indicate mammalian lineage-specific regulation of HOX genes that could be responsible for species phenotypic differences.

### HOX gene patterning in the limb

Macropodid marsupials have very specialized limbs. The forelimb is developed at birth to allow the neonate to climb to the pouch to locate and attach to one of the four available teats [123] but the hind limb, which eventually becomes the dominant feature of this hopping family, is barely formed at birth. Despite its embryonic nature, it is already possible to see the syndactylus arrangement of digits in which digits 2 and 3 are fused, digit 4 is enlarged and digit 5 is reduced. HOX genes play an important role in this arrangement. In particular, *HOXA13 *and *HOXD13 *play essential roles in digit development (reviewed in [119]). *HOXA13 *and *HOXD13 *in the developing tammar limb have both a conserved and divergent expression pattern (KY Chew, H Yu, AJ Pask, G Shaw, and MB Renfree, unpublished results). Tammar HOXA13 has a transient expression compared to the chicken and mouse, while tammar *HOXD13 *is expressed in distal limb elements, as in other vertebrate species [124,125]. Early differences in the expression pattern were observed in the specialized tammar hindlimb compared to other species. These subtle differences could direct the morphological specialization of the tammar hindlimb to allow for the hopping mode of locomotion.

### Pre-natal growth and placental genes

Mammals require genes that regulate growth both pre- and postnatally. Genes of the growth hormone/insulin-like growth factor-I (GH-IGF-I) axis are highly conserved in marsupials owing to their important function in pre- and postnatal growth. Sequencing and expression analysis of the GH receptor gene shows that exon 3, which is associated with variable growth and IGF-1 physiology in humans, is specific to the eutherian lineage and has undergone more rapid evolution in species with placental variants of GH and prolactin, indicating a possible fetal-specific role for the GH receptor in these species [126].

Prenatally, the placenta is a critical regulator of fetal growth. Genes involved in growth regulation in eutherian mammals (GH, GH receptor, prolactin, luteinizing hormone, IGF-1, IGF-2, insulin and their receptors) are all highly conserved in the tammar and all are expressed in the yolk sac placenta of the tammar wallaby, suggesting a conserved role for these hormones and growth factors during pregnancy in therian mammals [127]. GH and its receptor appear to be under tight regulation in the placenta, with expression increasing dramatically after close attachment of the placenta to the endometrium. Placental expression of both *GH *and *GHR *peaks at the end of pregnancy during the most rapid phase of fetal growth. These data indicate that GH and other pituitary hormones and growth factors are as essential for growth and development of the placenta in the tammar as in eutherian mammals.

Postnatally, maturation of GH-regulated growth in marsupials occurs during late lactation at a developmental stage equivalent to that of birth in precocial eutherian mammals (B Menzies, G Shaw, T Fletcher, AJ Pask, and MB Renfree, unpublished results) and it appears that this process is not associated with birth in mammals but instead with relative maturation of the young. This emphasizes the importance of nutrition in controlling early development in all mammals as they transition to independence. The neonatal tammar expresses ghrelin, a peptide that stimulates both hunger and GH release, in the stomach, ensuring that it can feed from a relatively early developmental stage [128].

## Genomic imprinting

Genomic imprinting is a widespread epigenetic phenomenon characterized by differential expression of alleles, depending on their parent of origin. Imprinted genes in eutherian mammals regulate many aspects of early growth and development, especially those occurring in the placenta. Most, but not all, genes that are imprinted in mouse and human have orthologues in the tammar genome; an exception is the Prader-Willi-Angelman syndrome region containing *SNRPN *and *UBE3A*, which does not exist in tammar, nor in monotremes, so was evidently recently constructed in eutherians by fusion and retrotransposition [129]. Some tammar orthologues of genes that are imprinted in eutherians are not imprinted [130,131]. So far the orthologues of 13 eutherian imprinted genes examined have a conserved expression in the marsupial placenta, but only 6 of these are imprinted in marsupials [132,133].

Marsupial orthologues of the classically imprinted IGF-2 receptor (*IGF2R*), insulin (*INS*) or paternally expressed gene 1/mesoderm specific transcript (*PEG1/*MEST) also show parent-of-origin expression in marsupials. However, some genes that are imprinted in eutherians, such as *Phlda2 *in the *KCNQ1 *domain, a negative regulator of placental growth, are not imprinted in the tammar [134]. This demonstrates that acquisition of genomic imprinting in the *KCNQ1 *domain occurred specifically in the eutherian lineage after the divergence of marsupials, even though imprinting of the adjacent *H19-IGF2 *domain [135] arose before the marsupial-eutherian split. A similar scenario applies to *DLK1*, *DIO3 *and *RTL1 *(*PEG11*), which are not imprinted in marsupials [130,136].

Differentially methylated regions (DMRs) are the most common signals controlling genomic imprinting in eutherian mammals. However, no DMRs were found near the tammar orthologues of the classically imprinted genes *IGF2R*, *INS *or *PEG1/MEST*, although these genes still showed parent of origin specific expression differences. Other marsupial imprinted genes (*H19*, *IGF2 *and *PEG10*) do have DMRs, indicating that this mechanism of gene control evolved in the common therian ancestor at least 140 million years ago [133]. Using comparisons with the tammar genome, we have been able to reconstruct the emergence of an imprinted gene - *PEG10 *[137]. *PEG10 *is derived from a retrotransposon of the suchi-ichi family and was inserted after the prototherian-therian mammal divergence. This demonstrates that retrotransposition can drive the evolution of an imprinted region with a DMR [137]. In contrast, another retrotransposed gene also of the suchi-ichi family, *SIRH12*, has been identified specifically in the tammar genome but is not seen in eutherians. It appears to be tammar-specific since it is absent from the opossum genome. Its imprint status has yet to be ascertained [138].

The insulator genes *CTCF *(CCCTC-binding factor) and its paralogue *BORIS *(brother of regulator of imprinted sites) have orthologues in the tammar genome, and as in mouse, *CTCF *is expressed ubiquitously and *BORIS *is expressed in gonads. The existence of both genes in the monotreme and reptile genomes but the ubiquitous expression of *BORIS *in these species suggests that this gene became gonad-specific in therian mammals, coincident with the evolution of imprinting [139].

Although all imprinted genes so far identified in the mouse are expressed in the placenta, the few mouse genes that have been knocked out (for example, *Grb10*, *Peg3 *) that are also imprinted in the fetal brain have marked behavioral effects [140]. We now know that there are additional autosomal genes in the cortex and hypothalamus with sex-specific imprinting [141,142], so we can expect an increase in the identification of imprinted brain genes that influence behavior. Since a large proportion of known imprinted genes also have a role in postnatal growth and nutrient supply, and marsupials depend much more on lactation than most other mammals (see below), it is possible that genomic imprinting might function in the marsupial mammary gland as it does in the placenta. Transcription analysis has confirmed that two genes critical for the onset of lactation in the tammar, *IGF2 *and *INS*, are imprinted in the tammar mammary gland throughout the long period of lactation (JM Stringer, S Suzuki, G Shaw, AJ Pask, and MB Renfree, unpublished observations).

## Olfaction

### Vomeronasal organ

**Figure 8:**
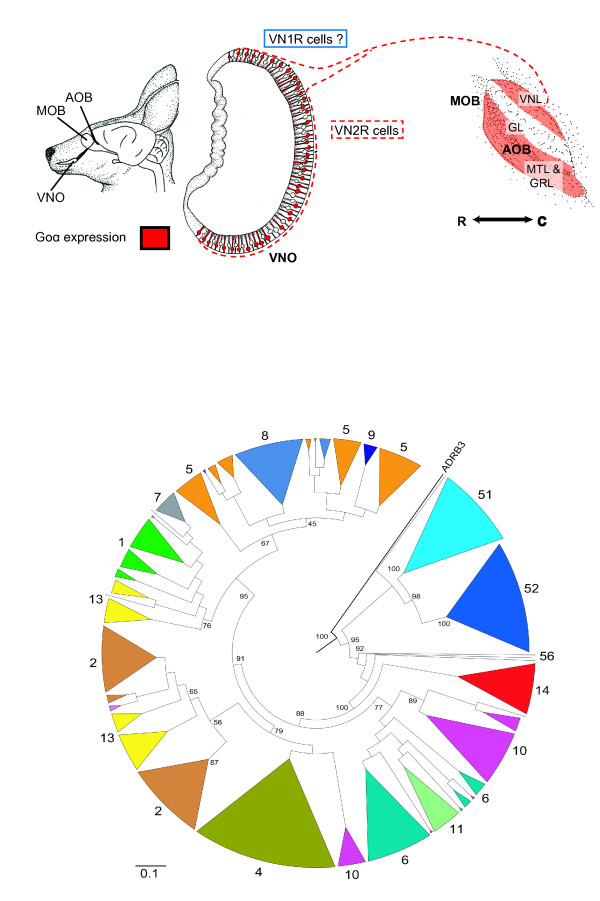
**Olfaction in the tammar**. **(a) **The olfactory apparatus of the tammar showing the pattern of vomeronasal receptor projections to the accessory olfactory bulb with the VN2 receptor cells (expressing Goα) projecting to all parts of the vomeronasal nerve layer (which may also be the case for the VN1 receptor cells (expressing Giα2). This projection pattern may reflect an intermediate type to the 'segregated type' and the 'uniform type' so far described. AOB, accessory olfactory bulb; GL, glomerular layer; GRL, granule cell layer; MOB, main olfactory bulb; MTL, mitral tufted cell layer; VNL, vomeronasal nerve layer; VNO, vomeronasal organ; VN1R and VN2R, vomeronasal receptors 1 and 2. **(b) **Olfactory receptor (OR) gene family in the tammar. The families of the OR gene repertoire. Neighbor joining tree of 456 full-length functional OR genes was rooted with opossum adrenergic β receptor. Only a few OR gene families (14, 51 and 52) have members that are most closely related to each other, whilst most other families have a high degree of relatedness to other families.

Pheromone detection in vertebrates is mostly mediated by the vomeronasal organ (VNO). The VNO organ is well developed in the tammar [123]. Pheromone detection occurs via two large families of vomeronasal receptors (VNRs). VN1Rs are associated with the protein Giα2 and VN2Rs with Goα using a signaling cascade dependent on transient receptor potential channel, subfamily C, member 2, encoded by the *TRPC2 *gene. Previous characterizations of *TRPC2 *in rodents led to confusion regarding its functionally relevant transcripts. Expression analysis and characterization of transcripts in the tammar have now shown that the locus consists of two distinct genes, one that is VNO-specific (*TRPC2 *proper) and a previously unidentified copy that is ubiquitously expressed (*XNDR*) [143]. *XNDR *has homology with *XRCC1*, suggesting a role in DNA base excision repair due to homology with *XRCC1 *[144]. *Giα2 *and *Goα *have high sequence conservation and both are expressed in the tammar VNO and accessory olfactory bulb (NY Schneider, G Shaw, PT Fletcher, and MB Renfree, unpublished results). The projection pattern of the tammar Giα2 and Goα expressing receptor cells differs from that of the goat (uniform type) and the mouse (segregated type) and so may represent a new intermediate type (Figure 8a), with Goα not being confined to the rostral or caudal part of the accessory olfactory bulb, respectively, but found throughout (for example, [145]). Immunostaining results further suggest that Giα2 may follow the same pattern, but confirmation awaits the availability of a more specific antibody.

### Olfactory receptor family genes

The marsupial genome has one of the largest mammalian olfactory receptor gene families, containing up to 1,500 olfactory receptor (OR) genes that apparently provide the tammar with a remarkably large range of odor detection in both the VNO and the main olfactory epithelium (A Mohammadi, H Patel, ML Delbridge, and JAM Graves, unpublished results) (Figure 8b). Certainly the neonate uses odor to locate the teat within the pouch [146]. There are 286 OR gene families in the tammar genome, with duplications especially in class I OR families OR8, -11, -13 and -51. However, the class II family OR14 has only one-third of the number found in the platypus genome, and eutherians have lost them altogether [147] (A Mohammadi, H Patel, ML Delbridge, and JAM Graves, unpublished results; Figure 8b). We found that class I OR families, particularly OR8, -11, -13 and -51, have undergone expansion in the tammar lineage, whereas the class II family OR14 has only one-third of the number found in the platypus genome and eutherians have lost them altogether [147].

The tammar and opossum have remarkably similar OR gene repertoires despite the significant variation in OR genes found in eutherian species that diverged about the same time. The OR genes are observed in gene clusters across all chromosomes, except chromosome 6 (Figure 2). The tammar Y chromosome has not yet been fully characterized but OR genes are not found on the Y of tammar or other mammals. They are found in the same conserved syntenic blocks as in the human (Figure 2) and opossum (data not shown), except clusters 9, 11 and 24, which have moved to 4q, and part of cluster 23, which is on 2q.

## Lactation

Lactation is a defining character of mammals [148,149]. This is especially true of marsupials that give birth to highly altricial young that depend upon milk for growth and development during a relatively long lactation period. The marsupial mother dramatically alters milk production and composition throughout lactation, specifically for each stage of development of the pouch young [26,150,151]. They are even able to produce milk of differing compositions from adjacent mammary glands, a phenomenon known as concurrent asynchronous lactation (reviewed in [152]).

Lactation in the tammar extends for approximately 300 days and is divided into 3 phases based upon the sucking pattern of the young (phase 1 (late pregnancy-birth), lactogenesis; phase 2A (day 0 to 100), permanently attached to the teat; phase 2B (day 100 to 200), intermittently sucking and confined to the pouch; phase 3 (day 200 to 300), in and out of the pouch), accompanied by changes in milk composition and mammary gland gene expression [26]. The tammar mammary gland transcriptome consists of two groups of genes [63]. One group is induced at parturition and expressed throughout lactation, as in eutherians. These genes include the milk protein genes encoding α-, β-, and κ-casein (*CSN1*, *CSN2 *and *CSN3*) and the α-lactalbumin (*LALBA*) and β-lactoglobulin (*LGB*) whey protein genes. However, the tammar genome lacks additional copies of α- or β-like caseins that are present in monotremes and eutherians (Figure S3 in Additional file 4).

The second group of mammary genes is expressed only during specific phases of lactation. This group includes marsupial-specific milk protein genes such as the late lactation proteins (*LLPA *and *LLPB*) as well as others such as whey acidic protein (*WAP*) [153] that are also found in milk of many eutherians [154] but lacking in humans, goat and ewe [155]. Evidence is now emerging that changes in composition of the major milk proteins and many bioactives [156,157] contribute to a more central role of milk in regulating development and function of the mammary gland [158] to provide protection from bacterial infection in the gut of the young and the mammary gland [159] (A Watt and KR Nicholas, unpublished results) and to deliver specific signals to the young that regulate growth and development of specific tissues such as the gut [160]. There is also a novel putative non-coding RNA (*PTNC-1*) expressed in the mammary gland throughout lactation. *PTNC-1 *is derived from a region of the genome that is highly conserved in mammals, suggesting it may have an important functional role [63]. Tammar *ELP *(early lactation protein), originally thought to be marsupial-specific (phase 2A) [63], has a eutherian orthologue, colostrum trypsin inhibitor (*CTI*), which is present in some eutherians but is reduced to a pseudogene in others (EA Pharo, AA De Leo, MB Renfree, and KR Nicholas, unpublished results). The *ELP*/*CTI *gene is flanked by single-copy genes that map to orthologous regions of the genome - strong evidence that *ELP/CTI *evolved from the same ancestral gene. *ELP*/*CTI *has not yet been detected in monotremes. Other marsupial-specific milk protein genes identified include trichosurin and the putative tammar milk proteins *PTMP-1 *and *PTMP-2 *[63]. Remarkably, the tammar *PTMP-1 *gene has been identified in the tammar genome sequence, but does not seem to occur in the genome sequence of the short-tail grey opossum. Thus, *PTMP-1 *may be macropodid-specific.

## Conclusions

The tammar, a small kangaroo species, is the model Australian marsupial that has played a particularly important role in the study of reproduction, development, immunity and the evolution of the mammalian sex chromosomes. Here, we have presented its genome sequence and associated resources, including transcriptome sequence data from a range of tissues. Together these data have provided new insights into a host of important gene families. We identified novel tammar-specific, as well as conserved but previously undiscovered, miRNAs that regulate the *HOX *genes, a novel SINE class that is rRNA-derived and a novel class of small RNAs. We show that there has been expansion of several gene families, especially of the MHC and OR genes, that there are features that are of specific importance to marsupials, such as the innovation of genes in lactation and the presence of genomic imprinting in the mammary gland. However, there is high conservation in testicular and ovarian genes, one of which, *DHH*, is only the second mammal-specific gonadal development gene so far identified. The Y chromosome is minute but relatively gene rich and conserved in marsupials. The X chromosome reflects the ancestral mammalian X and perhaps an ancestral stochastic dosage compensation that operates without an X chromosome inactivation center. These initial tammar genome analyses have already provided many unique insights into the evolution of the mammalian genome and highlight the importance of this emerging model system for understanding mammalian biology.

## Materials and methods

Materials and methods are briefly described in the body of the paper and extensively in the supplementary methods (Additional file 1).

## Data availability

Public database accessions are provided for all raw datasets where they are first mentioned in the text. The latest version of the genome assembly is available in NCBI under the GenBank accession ABQO000000000; Meug_1.1 has accession ABQO010000000; Meug_2.0 has accession ABQO020000000. All versions of the genome assembly are also accessible via the web [161].

## Abbreviations

BAC: bacterial artificial chromosome; BCM-HGSC: Baylor College of Medicine Human Genome Sequencing Center; bp: base pair; crasiRNA: centromere repeat-associated short interacting RNA; DHH: Desert hedgehog; DMR: differentially methylated region; EST: expressed sequence tag; GH: growth hormone; IFITM: interferon inducible transmembrane protein; IGF: insulin-like growth factor; KERV: kangaroo endogenous retrovirus; LINE: long interspersed nuclear element; LTR: long terminal repeat; MHC: major histocompatibility complex; miRNA: microRNA; NOR: nucleolar organizing region; OR: olfactory receptor; PGC: primordial germ cell; piRNA: Piwi-interacting RNA; SINE: short interspersed nuclear element; VNO: vomeronasal organ; VNR: vomeronasal receptor; WGS: whole-genome shotgun.

## Authors' contributions

All authors were members of the tammar wallaby genome sequencing consortium. Authors contributed to sequencing, assembly, analysis, experiments or writing as follows (team leaders are in bold). Joint lead authors: **MBR, ATP**. Principal investigators: JAMG, SMF, RAG, DWC, MBR, TPS, AF. Tammar genome size: **AJP, WR**, RJO'N, KCW, MFS. Physical and linkage mapping: **JED**, CW, FWN, KRZ, JAMG. Highly divergent or tammar-specific transcripts: **AJP**, AF, TH, YK, HY, MBR. Transcriptome: **ATP, AF**, AJP, MBR, YK, Z-PF, YSuz, SS, AT, YSak, SK, YN, ST. Genebuild: **SMJS**, SF. Sequence conservation and gene family expansion: **PF, ESWW**, KBl, JH. GC content: **WR, RJO'N**, JL. Analysis of Repeats and small RNAs: **RJO'N**, JL, DMC. Immunity: **KB**, ESWW, HVS, JED, MBR, KRS, CW, JW, BGC, ATP. Sex chromosomes: **PDW, JAMG**, JED. X chromosome inactivation: **JAMG, PDW**, JED, SAN. Reproductive genes: **AJP, MBR, GS**, DH, WO'H, YH. Developmental genes: **MBR, AJP**, GS, SRF, HY, K-YC, BRM, RJO'N, JL. Genomic imprinting: **MBR, AJP, JAMG**, GS, JS, SS, TAH. Olfaction: **MBR, JAMG**, SRF, AM, MLD, NYS, GS. Lactation: **CML, KRN**, EAP. Additional bioinformatics contributions: AH, BL, KAM, MJW. Australian Genome Research Facility Sanger sequencing: **SMF**, EK, AMG, PW, AM, JE, CT, DT, AS, LY, TL, MH-R, AH, JD, DW, SW, YSun. HGSC leadership: KCW, DMM, RAG. HGSC Sanger production: SNJ, LRL, MBM, GOO, SJR, JS, LN, AC, GF, CLK, HHD, VJ. HGSC SOLiD production team: HHD, CJ. HGSC 454 production: CLK, FL, RT. HGSC genome assembly and analysis: LC, JD, YL, JYS, X-ZS, GW, KCW. UConn sequencing and assembly improvement (Meug_2): **RJO'N, AJP**, JL, TH, IM. Senior authors: SMF, JAMG, RJO'N, AJP, KCW. Senior authors contributed equally and principal investigators contributed equally.

## Supplementary Material

Additional file 1 **Supplementary material**. Supplementary materials and methods, results and tables [39,42,46,47,58,74,164-192]. Click here for file

Additional file 2 **Figure S1 - comparison of gene sizes in *Monodelphis domestica *and *Macropus eugenii***. One-to-one opossum orthologues of tammar genes located more than 1 kb from the end of a scaffold were downloaded from Ensembl v62. The genomic lengths of the genes are plotted as a scatter plot on the log_2 _scale. A 1:1 linear relationship between gene sizes is present for genes less than the average scaffold size, suggesting that no major change in genome size has occurred in genic regions. A trend towards larger genes in opossum with log_2 _length > 15 is driven primarily by incompleteness of tammar genes when the gene size is larger than the average scaffold size. Click here for file

Additional file 3 **Figure S2 - analysis of the alignment of transcriptomic reads from different tissues to the tammar genome**. **(a) **Proportion of reads that align to unannotated regions, annotated genes, within 2 kb upstream or downstream of a gene, or fail to align to the tammar genome. **(b) **Proportion of mapped reads that align to unannotated regions, annotated genes, or within 2 kb upstream or downstream of a gene in the tammar genome. Click here for file

Additional file 4 **Figure S3 - Comparative analysis of the mammalian casein locus showing the expansion of the casein locus in mammals**. Comparison of the casein locus organization in the platypus, tammar, opossum, cattle, mouse and human genomes. Drawn to scale and aligned on the β-casein gene. Genes are represented by a box with a tail arrow pointing in the direction of gene transcription. Gene models for confirmed genes were generated from mammary gland EST data (platypus and tammar) or retrieved from Ensembl (others) when available. The tammar locus is not fully resolved and sequence scaffolds (indicated by black bars and scaffold numbers) have been aligned with the opossum sequence. Gaps in the tammar genome mainly fall in regions containing a repeated transposon type I in the opossum (black arrows), probably compounding the assembly of the tammar genome. Blank boxes represent putative genes based on similarity, grey boxes represent genes with observed expression. Note the close proximity of α- (*CSN1*, csna) and β- (*CSN2*, csnb) casein genes in reverse orientation on the left and the expansion of the region between β- and kappa- (*CSN3*, csnk) casein on the right. Except for β-casein, all genes are transcribed from left to right. In monotremes, a recent duplication of *CSN2 *has led to *CSN2b*, whereas in eutherians, an ancient duplication produced *CSN1S2*, which has been duplicated in some species to produce *CSN1S2b*, now a pseudogene in human but not in mouse. In the marsupial locus, there is no casein duplication and the spacing region contains several copies of an invading repetitive element (black arrows), suggesting active rearrangement of this region in the ancient marsupial lineage, probably resulting in the deletion of a putative ancient casein duplicate in the area. Click here for file
