# Supplementary material for: Genome sequence of an Australian kangaroo, Macropus eugenii, provides insight into the evolution of mammalian reproduction and development
Source: Genome Biol. 2011 Aug 19;12(8):R81. doi: 10.1186/gb-2011-12-8-r81 (PMC3277949; doi:10.1186/gb-2011-12-8-r81)
Supplement: Additional file 1 — Supplementary material. Supplementary materials and methods, results and tables [39,42,46,47,58,74,164-192]. [file gb-2011-12-8-r81-S1.doc]

**Genome sequence of an Australian kangaroo, *Macropus eugenii*, provides insight into the evolution of mammalian reproduction and development**

**SUPPLEMENTARY MATERIAL**

**Genome sequencing**

DNA purification and sequencing reactions (standard AB fluorescent Sanger sequencing technology) were performed using automated solid phase reversible immobilization (SPRI) technology for processing of the DNA preps (SprintPrep Kit, Agencourt Bioscience Corporation) and the sequencing reactions (CleanSeq Kit, Agencourt Bioscience Corporation) [164].

**Construction and end-sequencing of tammar large-insert genomic DNA libraries.**

A bacterial artificial chromosome (BAC) library, named as MEB1, was constructed from DNA isolated from a male tammar wallaby cell line. In brief, genomic Sac I-digested DNA fragments were inserted to the BAC vector pKS145 [165]. The X chromosome enriched fosmid library, named as MEFX, was constructed from DNA isolated from flow-sorted X chromosomes of a female tammar wallaby cell line, #2070. The extracted DNA molecules were physically fragmented and inserted to the fosmid vector pKS150 [166]. In total, 74,381 MEB1 clones and 17,706 MEFX clones were end-sequenced. The DDBJ accession numbers were DE842016-DE989327 for MEB1 and DH430751-DH462000 for MEFX.

**Genome assembly**

The Meug_1.0 assembly was produced by assembling whole genome shotgun reads with the Atlas genome assembly system at the Baylor College of Medicine Human Genome Sequencing Center [47]. About 10 million reads were presented to the assembler and 8.5 million reads were assembled, representing about 6.8 Gb of sequence and about 2x sequence coverage of the (clonable) tammar genome (using a 3.6 Gb estimated genome size: see Tables S1-S3). The clone coverage was estimated to be approximately 4x, based on the sum of insert sizes for paired reads divided by the estimated genome size of 3.6 Gb.

The 9.7 million reads were screened using a Cross_match [167] comparison to known sequencing and cloning vectors with the following parameters (coarse screen parameters of minmatch = 20, penalty = -2, and minscore = 30; and finescreen parameters of minmatch = 12, penalty = 2 and minscore = 20). The reads were trimmed with Atlas-screen-window with minimum of 50 contiguous good bases (-l 50), and 40 bases window (-w 40) with  quality scores >= 20 (-q 20).

The overlaps among the 9.6 million trimmed reads were assessed using the Atlas-overlapper [47] with the following parameters (-b 0 -e 30000 -B 10 -R 150 -H 151 -k 15  -Y 150  -m 6  -M 50 -S 199 -I -G 8 -p 103058537). Highly repetitive reads were defined as reads that had more than 22 reads that shared sequence overlaps or reads that overlapped with reads that had more than 90 reads that shared sequence overlaps.

Trimmed reads that clustered into groups of two or more that were not highly repetitive, reads were assembled by group using Phrap with the (-ace -forcelevel 10) parameters [168].

The tammar assembly process incorporated two additional steps that were not in the published methods [47].  These steps include the removal of highly repetitive reads using the criteria described above and the application of Atlas-bettergraph. Atlas-bettergraph attempts to removing "forking" overlaps at the potential repeat boundaries with these parameters (d=0.4 and p=0.5).

The assembled sequences were linearized using Cross_match (minscore = 100) to detect and merge overlapping sequences between neighboring contigs within scaffolds for overlapping tails shorter than 100 bp.

Reads that could not be clustered by the Atlas Overlapper into groups of 2 or more were not assembled. The N50 of the contigs is 2.5 kb and the N50 of the scaffolds is 16.05 kb for the Sanger Meug_1.0 assembly. The N50 size is the length such that 50% of the assembled genome lies in blocks of the N50 size or longer. The total length of all contigs for the Sanger Meug_1.0 assembly is 2.55 Gb. When the gaps between contigs in scaffolds are included, the total span of the assembly is 2.945 Gb.

Highly repetitive reads were defined as reads that had more than 22 reads that shared sequence overlaps or reads that overlapped with reads that had more than 90 reads that shared sequence overlaps. These reads were assembled separately to generate the repetitive contigs for the genome.

Following the Sanger Meug_1.0 sequence assembly, the genome was further sequenced using the ABI SOLiD technology (Table S4) to upgrade the genome assembly by additional superscaffolding. Only about 15 percent of the data, the mate-pairs where both ends are uniquely mapped, was used. Of these mates, about one third had the potential to bridge between scaffolds and two thirds were within a single scaffold. There were few errors in the existing Sanger Meug_1.0 assembly as indicated by the assessment of consistency of the mate-pairs that mapped within scaffolds. About 0.03 percent of these mates were mis-oriented and about one-tenth of one percent had an inter-mate-pair distance greater than 5kb. Using these data, a large number of scaffolds were merged to form new scaffolds and a small number of contigs were removed due to redundancy. The assembly statistics in Table S5 reflect these changes.

The completeness of the assembly was assessed by comparison to the available cDNA data. Using 758,062 454 FLX cDNAs sequences, 76 percent are found to some extent in the assembly and 30 percent are found with more than 80% of their length represented. See Table S6 for statistics. Compared to 14,878 ESTs from Sanger data, more than 85% are found in the assembly with at least one half their length aligned. See Table S7 for data.

**Assembly improvement method**

The first step in the assembly improvement pipeline is to map the paired reads against the draft contigs using appropriate mapping tools. Bowtie [169] was chosen as the mapping tool for the Illumina data. It was run with default parameters, except each read was allowed up to 3 mismatches total. The longer 454 reads were mapped with lastz [170, 171] with suggested parameters for mapping 454 data. The main difference between the two strategies is the longer 454 reads are better aligned with a tool that allows gapped alignment, Bowtie does not provide this feature and also has hard coded read size limits. The mapping results were filtered to only retain pairs for which both reads in a pair uniquely mapped, or one mate mapped uniquely and the other did not map at all. The uniqueness criterion ensures that the local re-assembly will not attempt to assemble repeated regions.

Next all unique short read pairs associated with a particular set of contigs, along with the draft contigs were reassembled using PHRAP [168] with default parameters. The minimum length of an overlap between two sequences was 14 base pairs, and the minimum score for an alignment was set to 30. Although there are many assembly tools available, PHRAP was found to fit best into this pipeline because it works with reads of different length. It should be noted that the assembler is unaware it is performing a local assembly, and was not designed to fully utilize paired information of short reads to determine order, orientation and position of the contigs. Therefore if the order of the new contigs differed from the order of original contigs, the offending contigs were replaced with their unaltered counterparts. This quality assurance step ensures the structure of the 1.2 assembly is consistent with the 1.1 assembly. It is our assumption that the draft contigs were ordered and oriented correctly since that process was done with the SOLiD reads which had the highest coverage.

The final step is to estimate the gap sizes based on mate pairs which span two adjacent contigs using an expectation maximization algorithm. There are several methods to estimate the size of a gap between consecutive contigs. Mate-pairs and pair-end reads mapped on the contigs form links between them, which can be generally referred to as edges. These edges have some statistical characteristics, such as insertion length and a standard deviation that are a result of the library preparation kit, method and operator error. This information can be used to estimate the gap size. One scaffolding tool, Bambus [42], brings the contigs to the midpoint of the range defined by the length constraints on the edges that linked the contigs. Another, Velvet [172], followed the method in [173], which first initialize the gap length to the mean of all edge insertion lengths. Starting from this value, it then bundles all compatible edges together and uses the mean insertion size as an estimation of the gap length. It would seem that using the mean is a natural way of estimating the gap lengths, however in a draft genome with many potentially large gaps between short contigs, there will exist a bias in the observed pairs mapped. As a result the mean of the observed pairs is usually not the right basis for gap estimation. The issue is resolved in our method through sampling.

The gap between two contigs is estimated using an expectation maximization algorithm. The max maximization step is to compute the gap estimate *x*, letting the mean insertion length of *N* pairs equal *u*, the initial value is the library average

$x=\frac{1}{N}\sum_{i=1}^{N} (u-d_{i1}-d_{i2})$.

Here, *d* is the distance from the position of the mapped read to the appropriate edge of the contig. The sampling step is as follows; given x, and the length of contigs, sample *u* from

completely mapped reads spanning the gap

$\sum_{i=1}^{N} (d_{i1}+x+d_{i2}-u)^{2}$.

These steps are repeated until the estimates converge. Additionally since multiple libraries were utilized the gap size estimates of two or more libraries was combined according to the following formula:

$$b(e)=\frac{\frac{n_{1}}{S^{2_{1}}}e_{1}+\frac{n_{2}}{S^{2_{2}}}e_{2}}{\frac{n_{1}}{S^{2_{1}}}+\frac{n_{2}}{S^{2_{2}}}}$$

Here, *b* is the bundled estimate, *S* is the standard deviation for particular library, and *n* is the number of pairs from that library.

The results were checked by PCR and a comparison of mapping statistics. In order to choose contigs to verify using PCR, the total pool of altered contigs was filtered to only include altered contigs with a gap size of less than 2 kb. From this set of contigs, 10 were randomly selected, 3 of which were triplets. Primers were designed on the left and right most contigs and it was verified that a complete extension occurred. Additionally a set of cDNA was downloaded from ENSEMBL as was a set of fully and partially sequenced BACs from NCBI for *Macropus eugenii*. These were mapped against the initial and improved assemblies and it was found that more bases and sequences were mapped against the improved assembly.

The paired read coverage is the number of pairs that mapped to contigs within 3 standard deviations of the expected insert size. This is indicative of the integrity of changes made to the contigs during the assembly update. There was almost a 2-fold improvement in paired read coverage between the assemblies (Table S8). It should be noted that the Illumina paired read coverage is very low as the libraries were generated with loose control over insert size selection. Table S9 shows the accuracy of changes made to the contigs between assemblies Meug_1.1 and Meug_2.0. Meug_2.0 recovered more of the high confident BACs and transcripts compared to the previous assembly.

**Estimation of genome size**

*Direct DNA content assessment*

Genome size was determined using quantitative PCR, according to the method described by [46]. Human (3 Gb) and *Saccharomyces cerevisiae* (12.1 Mb) genomes were used as controls to assess the accuracy of the assay and percentage error rate in the calculations. Tammar tissue was obtained from the same animal used to generate the genome. High molecular weight human genomic DNA was obtained from Invitrogen. DNA was extracted from the tammar tissue according to standard methods [174]. Real-time PCR was conducted using primers listed in Table S10. The single copy gene target in the tammar was *Dhh* as it has been shown to be single copy by Southern blot, fluorescent *in situ* hybridization, PCR and based on the initial genome assembly (1.0) and sequence trace archives (O'Hara, W.A., Azar, W.J., Behringer, R.R., Renfree, M.B., and Pask, A.J., unpublished results). The assay was performed on undiluted DNA, 1:10 and 1:100 dilutions to ensure accuracy and validate results. Both human (of similar size to the tammar genome) and *S.cerevisiae* (three orders of magnitude smaller) genomes were calculated to be within 8% and 4% of their actual size respectively, validating the technique (Table S11).

The tammar genome size assessment was carried out a total of 14 independent times over three separate real time plates and on two independent DNA extractions. The genome size estimates were extremely consistent across replicates and the tammar haploid genome size was calculated to be 2.7 Gb ± 170 Mb with a weight of approximately 2.96 pg, slightly smaller than that of human and mouse.

*Cytometry*

Tammar and opossum chromosome sizes were determined by flow cytometry in two different laboratories, RIKEN and Cambridge. At Cambridge, bivariate flow karyotyping was performed as previously described [175-177]. Chromosome preparations of tammar and human, or opossum and human, were measured together and separately but sequentially with the same flow cytometry settings, allowing a direct comparison between the tammar chromosomes and human chromosomes, and opossum chromosomes and human chromosomes. The “DNA-line” in the flow karyotypes was drawn from the origin through human chromosome 4. Each human, tammar, or opossum chromosome peak was projected onto this line, and the distance from the origin to this projection was an estimate of the DNA content of that particular chromosome. The established human chromosome sizes [178] were used as references for the tammar and opossum chromosomes taking into account both conversion and offset. Tammar and opossum genome size was determined by adding up the chromosome sizes.

The tammar chromosome complement consists of seven autosome pairs and one pair of sex chromosomes, X and Y. Chromosome 1 is relative large, chromosome 2 to 6 are medium sized, and chromosome 7 and X are smaller sized. Chromosome Y is a relative tiny chromosome. The sizes are presented in Table S12. Tammar chromosomes 1-6 are all larger than human chromosomes. For instance, MEU1 is about twice the size of HSA1. Notable is the size of MEUX of 150 Mb, which is a value between HSA8 and HSAX. Despite MEUX having a much smaller gene content than HSAX and being regarded as the ancestral therian X, its DNA content is comparable with HSAX, due to the addition of repetitive sequences of this NOR-bearing chromosome. The opossum chromosome X does not carry the NOR and has a size of 97 Mb (Table S12), which is about the expected 2/3 size of the eutherian chromosome X. By comparison of the DNA content and GC content of the tammar X and opossum X (Table S12) and assuming that the remainder part of the tammar X is conserved between tammar and opossum, one can calculate that the region added to the tammar X consisted of around 10 Mb GC and 43 Mb AT. The tammar genome size from the flow sorted chromosomes is estimated to be 2.457 Gb determined by adding up the chromosome sizes given in Table 1. This physical size is smaller than the human genome size of 3.08 Gb.

The karyotype of the South American *Monodelphis domestica* consists of eight autosome pairs and one pair of X-Y sex chromosomes. The chromosomes in the karyotype are ordered according to [39, 179]. Chromosome 1 is relatively large, chromosome 2 to 8 are all medium sized, only chromosome X is smaller sized. Chromosome 5 and 8 have similar sizes as chromosomes were ordered by centromere position as first parameter [179]. Chromosome Y is a tiny chromosome and too small to be measured by flow cytometry. Apart from MDO7 and MDOX, all MDO chromosomes are larger than human chromosomes. MDOX is much smaller than the human X (about two third), which is due to autosomal genomic regions being translocated to the eutherian X during evolution. The opossum genome size is estimated to be 3.172 Gb determined by adding up the chromosome sizes given in Table S12. This physical size is similar to the human genome size of 3.08 Gb, but bigger than the tammar genome size of 2.457 Gb.

Flow sorting genome size estimation of tammar chromosomes at RIKEN was performed using a standard flow-cytometry protocol (Cycle TEST PLUS DNA Reagent Kit, Becton & Dickinson). Nuclei fractions were isolated from cultured cells derived from three different tammar individuals, including the animal used for the genome sequencing and from two human cell lines, #GM130B, #GM18940, and stained with propidium di-iodide. The estimated tammar genome size was 3.6 Gb in average given that of human genome was 3.0 Gb. The exact values were 3.73, 3.59 and 3.65 Gb for the cell lines #2409♂, #3469♂ and #2070♀, respectively (see Table S13).

*Sequencing-based genome size estimation*

Finally, the genome size was estimated from the Sanger WGS sequence data using the Atlas-Genometer. This method is based on the Lander-Waterman model [180] and a linear model to relate the number of reads that have no overlaps to the observed number of these reads adjusting for repeats and sequencing errors. The method calculates the genome size based upon sampling subsets of reads from low coverage data samples and uses an Expectation Maximization algorithm to calculate a convergent genome size. Using a variety of Atlas-overlapper calculations [47] with between 360,000 and 3,600,000 reads, and initial genome sizes of 3.2 to 4.9 Gb, the tammar genome size is estimated to be 3.65 Gb for calculations that converge (smaller starting genome sizes did not converge) (Table S13). The method computes a value of 3.07 Gb for the human genome (3.08 Gb including Ns, 2.85 Gb excluding Ns for NCBI build 36) using 0.5x 454 data. Assuming that the estimate is within 10% of the actual genome size, the range of estimated genome sizes for the tammar would be 3.29 to 4.1 Gb. Based on the available EST data and comparisons to the Meug_1.0 and Meug_1.1 genome assemblies (Ensembl), the genome size is estimated to be between 3.00 and 3.78 Gb. This calculation uses the fraction of the ESTs that are not aligned over at least 50% of their length in the existing assemblies to calculate the missing fraction of the genome. The lower number is based upon comparison to 14,878 Sanger ESTs, and the higher number is based upon comparison to 758,062 454 FLX ESTs.

*Tammar and opossum GC-content*

The GC content of each tammar or opossum chromosomes was determined using the GC content of human chromosome 7 [181] and human chromosome 10 [182] as a reference. Each peak in the flow karyotypes was projected on the GC axis of the flow karyotype. The distance to the origin of these projections was regarded as a measure for the absolute GC content. The relative GC content of human chromosome 7 and 10 was converted into an absolute number using the DNA content values given by Ensembl. The calculated absolute GC content, taking into account both conversion and offset, of tammar and opossum chromosomes were converted into relative GC contents using the DNA content of these chromosomes calculated above. In tammar, the chromosome GC varies from a relatively low 27% (chr7) to 37% (chr1) with an average of 34% (Table S12). This is substantially lower that the GC for human (42%) and opossum (40%). The tammar X also has a GC content (34%) lower than that of the opossum X (42%). Thus, tammar chromosomes are relatively GC poor.

**Sequence conservation**

Analyses were performed on tammar assembly version 1.0. Ensembl genesets used for comparative analyses were from release 58. BlastZ-net pairwise alignment [183] was constructed between tammar and opossum.

**Transcriptome sequencing and analysis**

*Construction and analyses of full-length cDNA libraries*

Prof. Renfree (University of Melbourne) provided tissues from testis, ovary and hypothalamus cDNA sequencing. The full-length cDNA libraries were constructed by Prof. Sugano's group at University of Tokyo (120,000 clones in total). End sequencing was produced by RIKEN-GSC (Toyoda and Kuroki) and Prof. Kohara's group at National Institute of Genetics. Sequence data from three cDNA libraries were combined and processed by removing vectors, low-quality (average QV<=15) nucleotides and poly(A) sequences. The 5' sequences were clustered at ninety percent identity using the cd-hit-est program. Dr. Tatsumoto (RIKEN-GSC) performed data analysis and clustering.

The oligo-capped full-length cDNA libraries were constructed from RNAs extracted from adult tammar tissues as described previously [184]. The resulting cDNA libraries were named as METC for testis, MEOC for ovary, MEHC for hypothalamus, and MEGC for gravid uterus, respectively. According to the method, we estimated that the range of the insert size of the libraries as ~3 kb. From these libraries, we obtained 360,350 end-sequence reads in total for both 5' and 3' ends of the inserts (DDBJ MEGC: FY469875-FY560833. MEHC: FY560834-FY602565. MEOC: FY602565-FY644882. METC: FY644883-FY736474).

CDNA sequencing was also performed at BCM-HGSC using the 454 GS FLX platform for two thymus libraries (thoracic and cervical samples). These data can be found in the NCBI Sequence Read Archive under Experiment numbers SRX019249 and SRX019250.

Tissue-specific high-quality full-length cDNA libraries are invaluable for the future biology in the post genome-sequencing era. We constructed such libraries from testis, ovary, hypothalamus and gravid uterus using oligo-capping technology [184]. Clones from each library were subjected to end-sequencing to evaluate composition and complexity of each transcriptome (360,350 sequence reads in total). After clustering, the library from hypothalamus, named MEHC, showed highest complexity, whereas the one from ovary, named MEOC, was lowest, 44.3% and 18.8%, respectively (percentage ratio of the number of clusters against those of sequence-reads). The clusters cover 88.5% (MEHC) to 92.9% (MEOC) of the current genome assembly (Meug_1.1), the others fell in-between, indicating high quality of our libraries. We then looked for representative genes in each library within the Ref-seq database (refseq_rna(blastdb), Date: 2010.10.30, [185]). For example, homolog of *KLH10* (OMIM 608778) and *ODF1/2* (OMIM 182878) genes, both function in spermatogenesis and male fertility were found in the testis library, named METC. The number of the reads of these genes reached 4.3% and 3.6% of the entire reads having E value <= 1e-30, respectively. Similarly, the hypothalamus library, MEHC, was rich in tubulin family genes, 7.9%, and some hormone related genes such as *SST* (somatostatin; OMIM 182450), 1.8% (Tables S18a).

When the transcriptomic reads were assembled and aligned to the tammar genome, we found that of the 141,574 contigs and unassembled reads, 94,890 aligned to 15,043 (84%) Ensembl-predicted genes (Release 57) were supported. Among the 2,834 of Ensembl genes not supported by transcriptomic data, 60% were protein-coding genes, 20% pseudogenes, and 20% non-coding RNAs. Only 25,415 contigs aligned to unannotated regions of the assembly (>2kb from a predicted gene). Of these, 5,545 had high quality matches (E-value<10^-5^) to proteins in the NCBI Non-Redundant (NR) database.

**Repeats**

The repeat content of the tammar genome was assessed using RepeatMasker, RepeatModeler and *ab initio* repeat prediction programs. The Repbase database of consensus repeat sequences was used to identify repeats in the genome derived from known classes of elements (Table 2). RepeatModeler uses a variety of *ab initio* tools to identify repetitive sequences irrespective of known classes [58]. Unannotated repeats were further queried in the following specialized repeat class annotation tools; TEclass [186], LTR_STRUC [187], RTAnalyzer [188], MUST [189], LTR-FINDER [190]. The remaining putative *de novo* repeats were aligned to the Repbase repeat annotations using BLAST. Any *de novo* repeat with ≥50% identity and coverage was annotated as that specific Repbase element. All of the putative *de novo* repeats that could not be annotated were considered *bona fide, de novo* repeats. The results from the database and *de novo* RepeatMasker annotations were combined, and any overlapping annotations were merged if they were of the same class of repeat element. Overlapping repeats from different classes were reported; therefore each position in the genome may have more than one unique annotation.

**Small RNAs**

The small RNA sequences were processed to remove sequencing adapters then selected for the appropriate size range. The trimmed and size selected libraries were then mapped to the genome using Bowtie [169]. One mis-match was allowed and all valid alignments for each of the sequences were reported.

*Bona fide* hairpin structures were identified using the hairpin loop prediction tool SRNALOOP [191]. A 100 base pair window up and downstream of each valid micro RNA alignment was selected as input for each experiment. This process is computationally challenging and a pipeline was developed to perform this task on a computer cluster. Hairpin target locations are simply defined as all valid mapped micro RNA locations.

The miRBase [74] mature sequences were mapped using Bowtie allowing for 3 mismatches and the longer hairpin and sequences were mapped using the local alignment tool Lastz [170, 171], 75% identity was required for each hit.

ChIP-seq mapping was performed as per (Lindsay, J., Carone, D.M., Murchison, E., Hannon, G., Pask, A.J., Renfree, M.B. and O’Neill, R.J., unpublished results) and MACsPeak calls were obtained as per [192]. CrasiRNAs were mapped to the genome using bowtie as above.

**SUPPLEMENTARY TABLES**

**Table S1.** Sanger-assembled reads statistics for Meug_1.0

|  | Reads (million) | Bases (billion) |
| --- | --- | --- |
| Total Sequenced | 9.7 | NA |
| Trimmed | 9.6 | 7.7 |
| Assembled | 8.5 | 6.8 |
| Unassembled | 0.52 | 0.42 |
| Repeat | 0.6 | 0.48 |

**Table S2.** Summary of Sanger sequencing libraries

|  | Number of Libraries | Number of Reads |
| --- | --- | --- |
| 3kb | 29 | 3,654,977 |
| 4kb | 2 | 2,788,140 |
| 6.25 kb | 2 | 3,248,488 |

**Table S3.** Sanger sequencing libraries

| Library | Number of reads | Insert size* | Std.dev. |
| --- | --- | --- | --- |
| 2070LUS01 | 61,488 | 3,000 | 1,000 |
| 2070LUS03 | 124 | 3,000 | 1,000 |
| 2070LUS05 | 1,839 | 3,000 | 1,000 |
| 2070LUS07 | 954 | 3,000 | 1,000 |
| 2070LUS08 | 14,509 | 3,000 | 1,000 |
| 2070LUS09 | 7,391 | 3,000 | 1,000 |
| 2070LUS10 | 1,535 | 3,000 | 1,000 |
| 2070LUS11 | 22,286 | 3,000 | 1,000 |
| 2070LUS12 | 16,170 | 3,000 | 1,000 |
| 2070LUS14 | 641 | 3,000 | 1,000 |
| 2070LUS15 | 735 | 3,000 | 1,000 |
| 2070LUS17 | 40,730 | 3,000 | 1,000 |
| 2070LUS18 | 85,122 | 3,000 | 1,000 |
| 2070LUS19 | 122,240 | 3,000 | 1,000 |
| 2070LUS21 | 1,396 | 3,000 | 1,000 |
| 2070LUM20 | 673 | 3,000 | 1,000 |
| 2070LUM22 | 2,031 | 3,000 | 1,000 |
| 2070LUS23 | 1,352 | 3,000 | 1,000 |
| 2070LUS24 | 1,405 | 3,000 | 1,000 |
| 2070LUS25 | 99,869 | 3,000 | 1,000 |
| 2070LUS26 | 2,928 | 3,000 | 1,000 |
| 2070LUS27 | 52,134 | 3,000 | 1,000 |
| 2070LUS28 | 1,394,102 | 3,000 | 1,000 |
| 2070LUS29 | 20,716 | 3,000 | 1,000 |
| 2070LUS30 | 91,093 | 3,000 | 1,000 |
| 2070LUS31 | 249,513 | 3,000 | 1,000 |
| 2070LUS32 | 660,312 | 3,000 | 1,000 |
| 2070LUS33 | 636,348 | 3,000 | 1,000 |
| 2070LUS34 | 65,341 | 3,000 | 1,000 |
| AUWAP | 2,785,918 | 4,000 | 1,000 |
| AUWCP | 2,222 | 4,000 | 1,000 |
| AUWBP | 1,158,027 | 6,250 | 1,250 |
| AUWDP | 2,090,461 | 6,250 | 1,250 |

*Insert size is the targeted insert size for the library

**Table S4.** SOLiD read statistics

|  | F3 | R3 | Total |
| --- | --- | --- | --- |
| Raw reads | 356,241,796 | 357,736,722 | 713,978,518 |
| Uniquely Mapped Pairs | 52,995,899 | 52,995,899 | 105,991,798 |
| Percent of Raw Reads | 15 | 15 | 15 |
| Scaffold Bridge | 17,158,908 | 17,158,908 | 34,317,816 |
| Percent of Mapped Pairs | 32 | 32 | 32 |
| Within Scaffold | 35,836,991 | 35,836,991 | 71,673,982 |
| Percent of Mapped Pairs | 68 | 68 | 68 |
| Mis-oriented | 10,777 | 10,777 | 21,554 |
| Percent of Mapped Pairs | 0.0301 | 0.0301 | 0.0301 |
| Inter-pair distance > 5kb | 36,687 | 36,687 | 73,374 |
| Percent of Mapped Pairs | 0.1024 | 0.1024 | 0.1024 |

**Table S5.** Genome assembly statistics

|  |  | Number | N50(kb) | Bases+Gaps(Mb) | Bases(Mb) |
| --- | --- | --- | --- | --- | --- |
| Meug_1.0 | Contigs | 1,211,471 | 2.5 | 2,549 | 2,549 |
| Meug_1.1 | Contigs | 1,174,382 | 2.6 | 2,536 | 2,536 |
| Meug_1.0 | Scaffolds | 616,418 | 16.05 | 2,945 | 2,549 |
| Meug_1.1 | Scaffolds | 277,711 | 36.6 | 3,075 | 2,536 |

**Table S6.** Comparison of genome assembly to 454 FLX cDNA data

|  | Meug_1.0 | |  | Meug_1.1 | |
| --- | --- | --- | --- | --- | --- |
| Length of aligned cDNA | Number of Reads | Percentage |  | Number of Reads | Percentage |
| 100% | 5,248 | 0.69% |  | 5,280 | 0.70% |
| 95% | 9,652 | 1.27% |  | 9,722 | **1.28%** |
| 80% | 230,242 | 30.37% |  | 229,378 | 30.26% |
| 50% | 511,128 | 67.43% |  | 508,791 | 67.12% |
| >0% | 579,735 | 76.48% |  | 576,647 | 76.07% |
| Total cDNAs | 758,062 | 100.00% |  | 758,062 | 100.00% |

**Table S7.** Comparison of genome assembly to 14,878 ESTs

|  | Percent Aligning to Genome | |
| --- | --- | --- |
| Length of cDNA aligned | Meug_1.0 | Meug_1.1 |
| 100% | 25.40% | 25.50% |
| 95% | 50.00% | 50.40% |
| 80% | 71.30% | 72.20% |
| 50% | 84.80% | 85.80% |

**Table S8**. **Summary statistics of the tammar genome assemblies**.

| type | count | total bases | avg length | coverage (2.7GB) | paired read coverage Meug_1.1 | paired read coverage Meug_2 | Coverage Change |
| --- | --- | --- | --- | --- | --- | --- | --- |
| Sanger | 11745817 | 9,445,367,851 | 804 | 3.5 | n/a | n/a | n/a |
| 454 | 1719180 | 396,757,212 | 230 | 0.15 | 0.41750 | 0.46629 | 1.12 |
| SOLiD | 710427474 | 17,760,686,850 | 25 | 6.58 | 0.13556 | 0.23594 | 1.74 |
| Illumina | 271875064 | 27,187,506,400 | 100 | 10.07 | 0.00056 | 0.00097 | 1.7376 |

**Table S9. Comparison of Meug 1.1 and 2.0 genome assemblies.**

| pool | assembly | # sequences | # bases | # mapped seqs | # mapped bases |
| --- | --- | --- | --- | --- | --- |
| BACs | 1.1 | 169 | 1,269,736 | 35 | 831,033 |
| BACs | 2 | 169 | 1,269,736 | 35 | 836,798 |
| ESTs | 1.1 | 84,718 | 104,868,227 | 68,336 | 60,076,451 |
| ESTs | 2 | 84,718 | 104,868,227 | 68,518 | 60,676,841 |

**Table S10.** Primers used for genome size assessment by qPCR.

| Target Gene | Name | Sequence 5’ →3’ | Product  Size (bp) |
| --- | --- | --- | --- |
| Saccharomyces cerevisiae ribosomal protein S3 gene (rps3), GenBank accession no. U34347. | S.c. RPS3-F1 | CGCTGACGGTGTCTTCTAC | 382 |
|  | S.c. RPS3-R1 | CCAACCAAGACCGAAGTTAT |  |
|  | S.c. RPS3-F2 | CGGAAACAACAACTTCACAA | 172 |
|  | S.c. RPS3-R2 | GACAGCGGACAAACCA |  |
| Homo sapiens sapiens p53 tumor suppressor gene, GenBank accession no. X54156. | H.s. p53-F1 | CGGCGCACAGAGGAAGAGAAT | 342 |
|  | H.s. p53-R1 | TTCCTAGCACTGCCCAACA |  |
|  | H.s. p53-F2 | CAAATGCCCCAATTGCAGGTA | 125 |
|  | H.s. p53-R2 | GACTGGAAACTTTCCACTTG |  |
| Macropus eugenii desert hedgehog gene, Ensembl transcript ID ENSMEUG00000014181 | M.e. DHH F1 | CCTGGACCGAGACTTACAGC | 465 |
|  | M.e. DHH R1 | CAGCCCAGTAGTTCCTCTGC |  |
|  | M.e. DHH F2 | GAGCTGGGGACTCGGTACTT | 179 |
|  | M.e. DHH R2 | ATGAGCCCACTGGTGACTCT |  |

**Table S11.** Genome sizes estimated by qPCR.

| Target | Sample Concentration | Calibration curve | Amplified copies | C (pg) | Γ (bp) |
| --- | --- | --- | --- | --- | --- |
| Tammar Dhh | 58.415 ng/µl | Y = -3.9329× + 37.314  (R^2^ = 0.999) | 1.972 × 10^4^ ± 6% | 2.96 | 2.7 x 10^9^ |
| *S.cerevisiae*  rps3 | 7.776 ng/µl | Y = -3.4661x + 38.755  (R^2^ = 0.999) | 5.87 x 10^5^ ± 4% | 0.014 | 12.7 x 10^6^ |
| *H.sapien* p53 | 273 ng/µl | y = -3.6772x + 38.461  (R^2^ = 0.996) | 7.88 x 10^4^ ± 8% | 3.48 | 3.1 x 10^9^ |

**Table S12.** Tammar and opossum chromosome sizes estimated by flow-sorting.

| Tammar chromosome | Size (Mb) | GC | Opossum chromosome | Size (Mb) | GC |
| --- | --- | --- | --- | --- | --- |
| Chromosome 1 | 486 | 0.37 | Chromosome 1 | 615 | 0.42 |
| Chromosome 2 | 367 | 0.35 | Chromosome 2 | 472 | 0.40 |
| Chromosome 3 | 355 | 0.35 | Chromosome 3 | 472 | 0.40 |
| Chromosome 4 | 340 | 0.34 | Chromosome 4 | 406 | 0.41 |
| Chromosome 5 | 340 | 0.34 | Chromosome 5 | 297 | 0.39 |
| Chromosome 6 | 286 | 0.31 | Chromosome 6 | 272 | 0.40 |
| Chromosome 7 | 133 | 0.27 | Chromosome 7 | 243 | 0.34 |
|  |  |  | Chromosome 8 | 297 | 0.39 |
| Chromosome X | 150 | 0.34 | Chromosome X | 97 | 0.42 |
| *Total Genome Size* | 2457 | 0.34 |  | 3172 | 0.40 |

**Table S13.** Tammar genome sizes estimated by flow-sorting at RIKEN.

| Sample name | Estimated genome size (Gb) |
| --- | --- |
| Tammar♂ #2409 | 3.50 |
| Tammar♀ #2070 | 3.74 |
| Tammar♂ #3469 | 3.59 |
| Average | 3.61 |

**Table S14.** Sample size, non-overlap reads and resulting genome size estimates for Sanger WGS comparisons

| Total Reads | Non-overlaping reads | Genome size (bp) |
| --- | --- | --- |
| 360,000 | 292,354 | 3,696,864,843 |
| 720,000 | 500,119 | 3,683,969,866 |
| 1,080,000 | 649,020 | 3,613,898,638 |
| 1,440,000 | 750,921 | 3,628,158,993 |
| 1,800,000 | 820,449 | 3,624,589,468 |
| 2,160,000 | 858,833 | 3,646,632,509 |
| 2,520,000 | 880,001 | 3,653,505,638 |
| 2,880,000 | 881,118 | 3,672,731,370 |
| 3,240,000 | 872,580 | 3,694,813,340 |
| 3,600,000 | 855,625 | 3,655,653,421 |

**Table S15. Tammar-specific gene family expansions**

| Tammar Ensembl Gene ID | Associated Gene Name |
| --- | --- |
| ENSMEUG00000008632 | Cd9 |
| ENSMEUG00000007545 | Cd9 |
| ENSMEUG00000013079 | Cd9 |
| ENSMEUG00000011422 | Atp12a |
| ENSMEUG00000003357 | Atp12a |
| ENSMEUG00000006532 | Trmt2b |
| ENSMEUG00000004230 | Slc37a2 |
| ENSMEUG00000001778 | Slc37a2 |
| ENSMEUG00000014731 | Olfr270 |
| ENSMEUG00000002021 | Olfr270 |
| ENSMEUG00000002000 | Olfr270 |
| ENSMEUG00000005442 | Hba-x |
| ENSMEUG00000005434 | Hba-x |
| ENSMEUG00000003990 | Snapc5 |
| ENSMEUG00000008996 | Snapc5 |
| ENSMEUG00000005596 | H2-Oa |
| ENSMEUG00000014073 | H2-Oa |
| ENSMEUG00000010599 | H2-Oa |
| ENSMEUG00000003576 | H2-Oa |
| ENSMEUG00000015590 | Tmbim6 |
| ENSMEUG00000012022 | Tmbim6 |
| ENSMEUG00000014841 | Tbl1xr1 |
| ENSMEUG00000007721 | Tbl1xr1 |
| ENSMEUG00000005644 | Sept8 |
| ENSMEUG00000007024 | Sept8 |
| ENSMEUG00000013104 | Slc29a1 |
| ENSMEUG00000000898 | Slc29a1 |
| ENSMEUG00000010554 | Rfng |
| ENSMEUG00000010012 | Rfng |
| ENSMEUG00000000128 | Ccna2 |
| ENSMEUG00000008916 | Ccna2 |
| ENSMEUG00000010254 | 2310046K01Rik |
| ENSMEUG00000005866 | 2310046K01Rik |
| ENSMEUG00000015304 | 2310046K01Rik |
| ENSMEUG00000004730 | Ncbp2 |
| ENSMEUG00000008126 | Ncbp2 |
| ENSMEUG00000009125 | Net1 |
| ENSMEUG00000009296 | Net1 |
| ENSMEUG00000013967 | Ocrl |
| ENSMEUG00000002996 | Ocrl |
| ENSMEUG00000006516 | Mospd1 |
| ENSMEUG00000002880 | Mospd1 |
| ENSMEUG00000001573 | Rbm22 |
| ENSMEUG00000009348 | Rbm22 |
| ENSMEUG00000015569 | Olfr15 |
| ENSMEUG00000016279 | Olfr15 |
| ENSMEUG00000015565 | Olfr15 |
| ENSMEUG00000000279 | Olfr15 |
| ENSMEUG00000012279 | Smc6 |
| ENSMEUG00000001811 | Smc6 |
| ENSMEUG00000004750 | Fzr1 |
| ENSMEUG00000015522 | Fzr1 |
| ENSMEUG00000006976 | Fzr1 |
| ENSMEUG00000006899 | Fzr1 |
| ENSMEUG00000008590 | Casp3 |
| ENSMEUG00000014666 | Casp3 |
| ENSMEUG00000013544 | Casp3 |

**Table S16.** Tammar-opossum sequence conservation

| Chromosome | % opossum sequence aligned to tammar | % opossum gene sequence aligned to tammar | % opossum unannotated sequence aligned to tammar |
| --- | --- | --- | --- |
| 1 | 40.6 | 13.9 | 26.7 |
| 2 | 38.5 | 14.7 | 23.8 |
| 3 | 36.0 | 11.5 | 24.4 |
| 4 | 38.2 | 13.0 | 25.2 |
| 5 | 36.1 | 11.4 | 24.7 |
| 6 | 39.4 | 14.1 | 25.2 |
| 7 | 36.2 | 9.9 | 26.3 |
| 8 | 39.0 | 13.3 | 25.7 |
| X | 40.9 | 12.7 | 28.2 |

**Table S17. Summary statistics from transcriptome libraries**

(a) Overview of all libraries

| Tissue | Mammary gland | Ovary | Gravid  uterus | Testis | Hypothalamus | Thymus |
| --- | --- | --- | --- | --- | --- | --- |
| Number of reads | 14,836 | 84,790 | 184,781 | 100,863 | 119,542 | 758,062 |
| Length (bp) | 608 | 1135 | 1125 | 725 | 1115 | 183 |
| GC (%) | 45.47 | 46.40 | 46.89 | 49.33 | 44.50 | 44.53 |
| Bases Masked (%)^a^ | 8.7 | 2.4 | 3.6 | 5.02 | 7.15 | 31.1 |
| Valid for assembly^b^ | 13,595 | 84,154 | 182,280 | 96,986 | 114,688 | 379,695 |

^a^  by RepeatMasker

^b^  after pre-processing with Seqclean

(b) Hybrid assembly with MIRA3

| Number of reads input | 1,294,241 (491,703 Sanger cDNAs) |
| --- | --- |
| Number of reads assembled | 880,700 (68.0%) |
| Number of singlets | 20,422 |
| Number of contigs | 121,152 |
| Largest contig | 4908 |
| N50 contig size | 1389 |
| N90 contig size | 509 |
| N95 contig size | 239 |
| Max coverage (total) | 1383 |
| Max coverage (Sanger) | 1740 |
| Max coverage (454) | 1361 |
| Average consensus quality | 16 |
| Strong unresolved repeat positions (SRMc) | 58 |

**Table S18. Summary statistics from transcriptome libraries**

**(a) Sanger end-sequenced cDNA libraries**

**
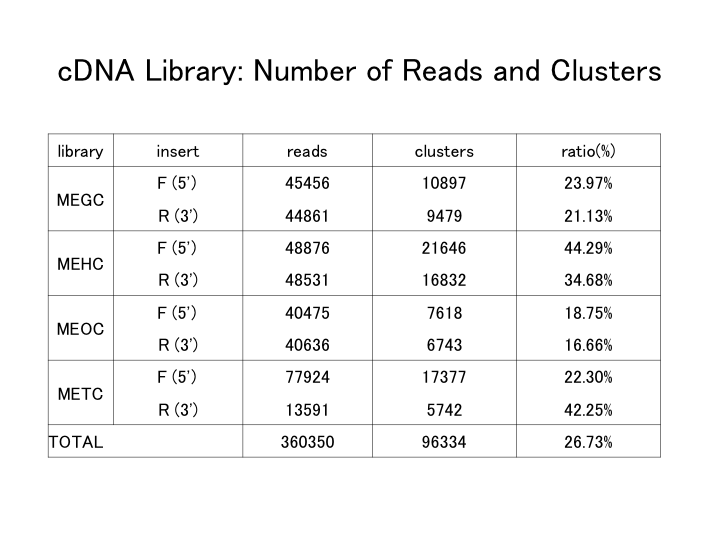
**

**(b) Hypothalamus top 20 most abundant hits in RefSeq**

**
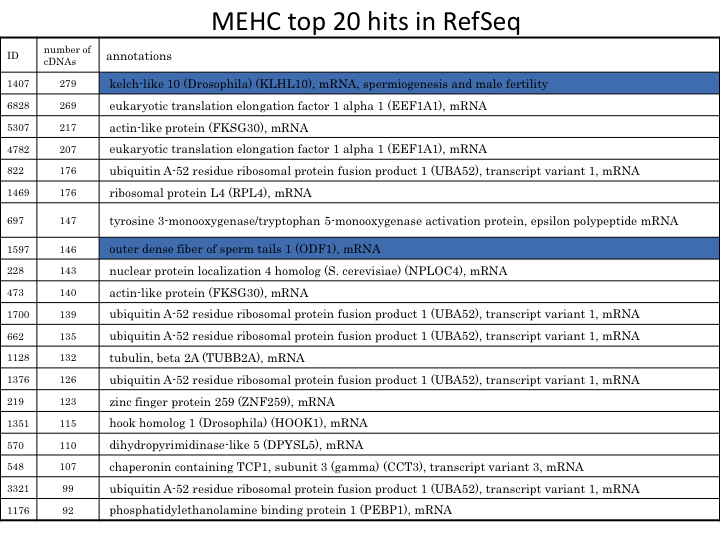
**
