# Supplementary figures and images for: Genome sequence of an Australian kangaroo, Macropus eugenii, provides insight into the evolution of mammalian reproduction and development
Source: Genome Biol. 2011 Aug 19;12(8):R81. doi: 10.1186/gb-2011-12-8-r81 (PMC3277949; doi:10.1186/gb-2011-12-8-r81)

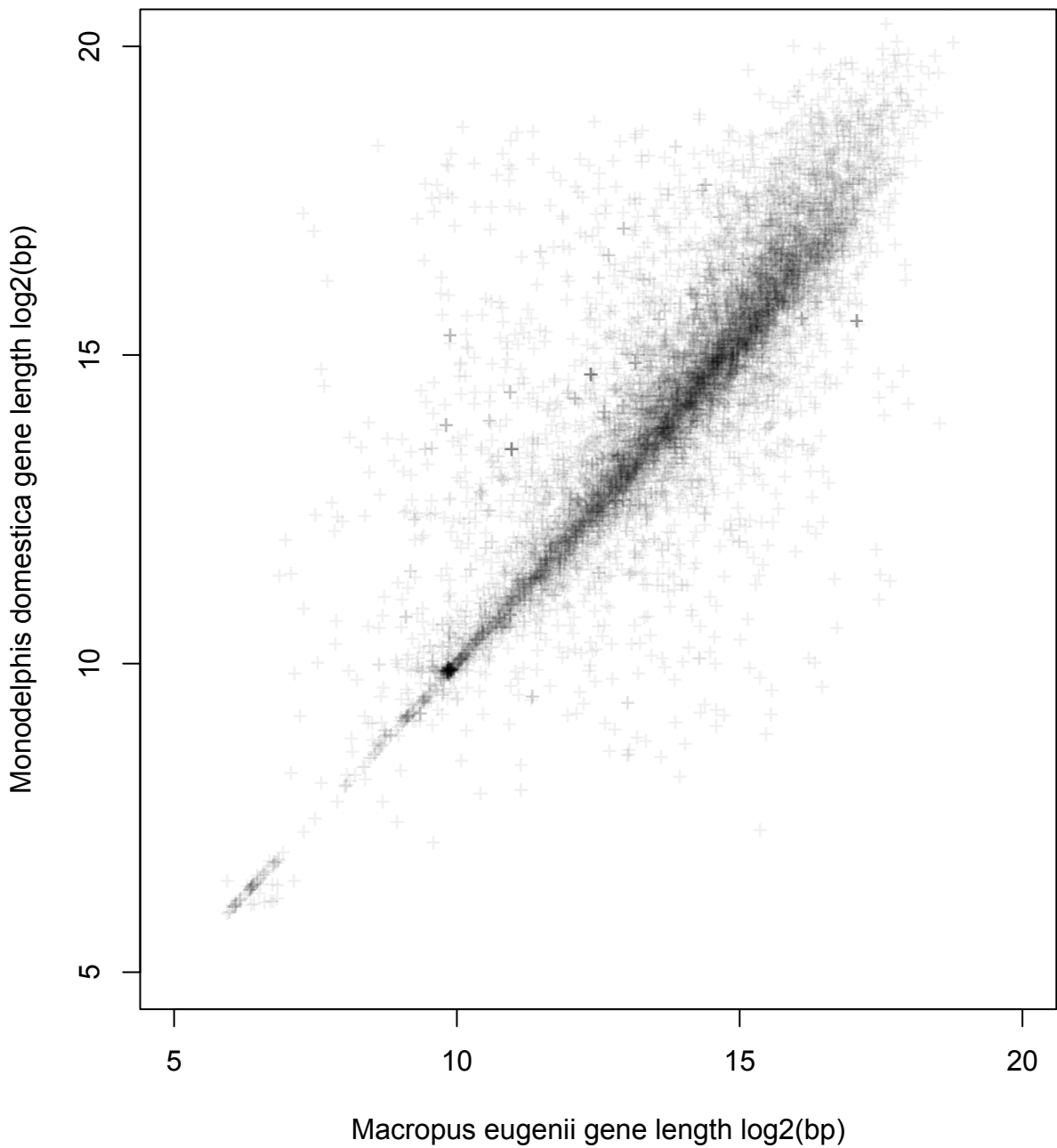

Supplement: Additional file 2 — Figure S1 - comparison of gene sizes in Monodelphis domestica and Macropus eugenii. One-to-one opossum orthologues of tammar genes located more than 1 kb from the end of a scaffold were downloaded from Ensembl v62. The genomic lengths of the genes are plotted as a scatter plot on the log2 scale. A 1:1 linear relationship between gene sizes is present for genes less than the average scaffold size, suggesting that no major change in genome size has occurred in genic regions. A trend towards larger genes in opossum with log2 length > 15 is driven primarily by incompleteness of tammar genes when the gene size is larger than the average scaffold size. [file gb-2011-12-8-r81-S2.pdf]

a.

Proportion of mapped reads

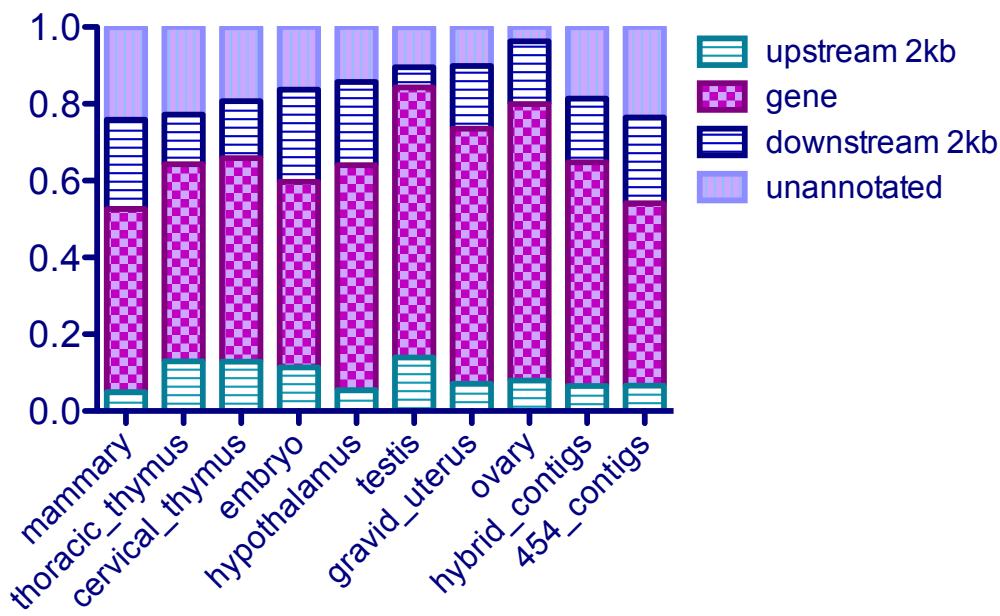

b.

Proportion of reads

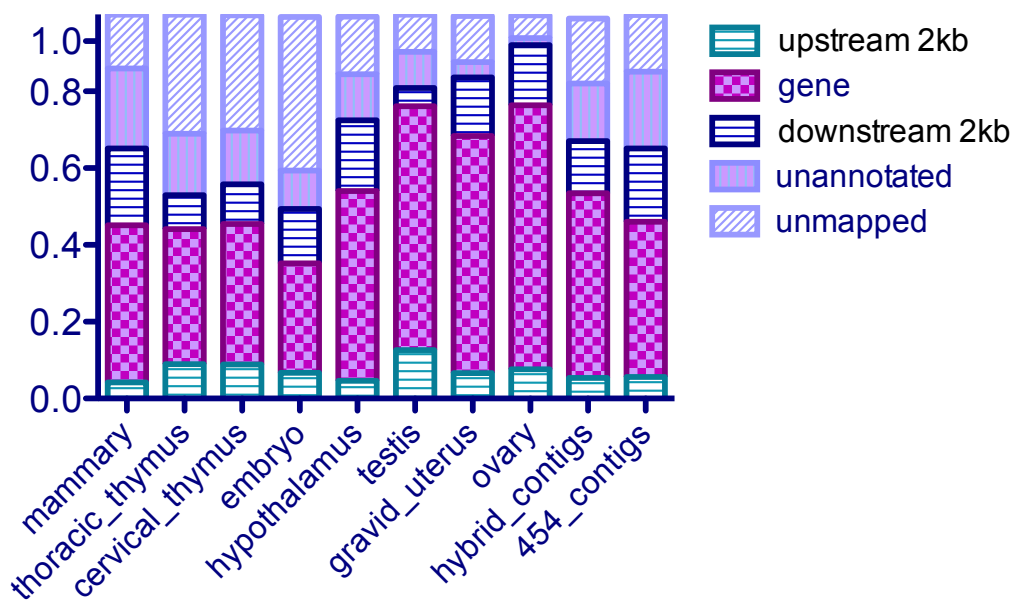

Supplement: Additional file 3 — Figure S2 - analysis of the alignment of transcriptomic reads from different tissues to the tammar genome. (a) Proportion of reads that align to unannotated regions, annotated genes, within 2 kb upstream or downstream of a gene, or fail to align to the tammar genome. (b) Proportion of mapped reads that align to unannotated regions, annotated genes, or within 2 kb upstream or downstream of a gene in the tammar genome. [file gb-2011-12-8-r81-S3.pdf]

Platypus ultra362

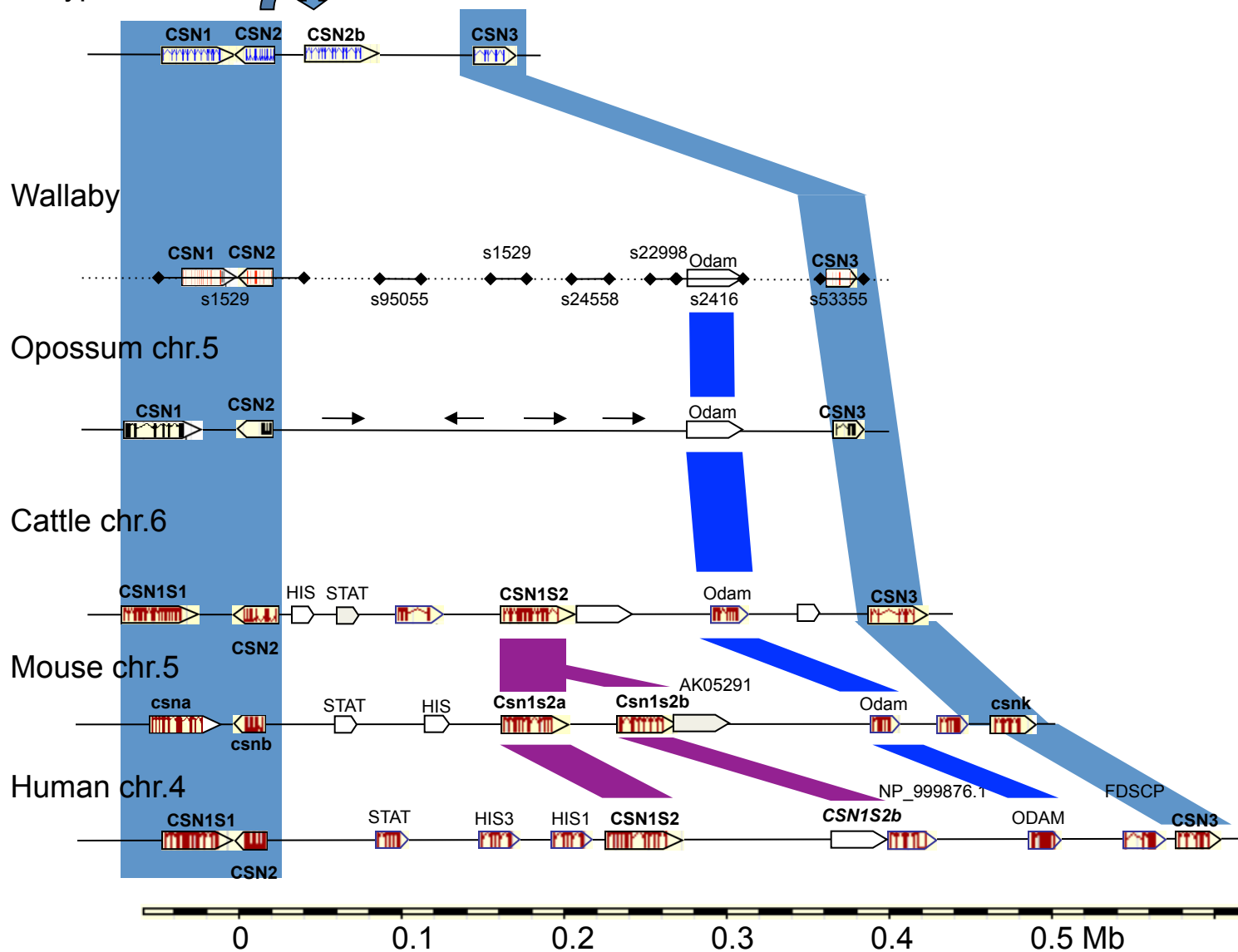

Supplement: Additional file 4 — Figure S3 - Comparative analysis of the mammalian casein locus showing the expansion of the casein locus in mammals. Comparison of the casein locus organization in the platypus, tammar, opossum, cattle, mouse and human genomes. Drawn to scale and aligned on the β-casein gene. Genes are represented by a box with a tail arrow pointing in the direction of gene transcription. Gene models for confirmed genes were generated from mammary gland EST data (platypus and tammar) or retrieved from Ensembl (others) when available. The tammar locus is not fully resolved and sequence scaffolds (indicated by black bars and scaffold numbers) have been aligned with the opossum sequence. Gaps in the tammar genome mainly fall in regions containing a repeated transposon type I in the opossum (black arrows), probably compounding the assembly of the tammar genome. Blank boxes represent putative genes based on similarity, grey boxes represent genes with observed expression. Note the close proximity of α- (CSN1, csna) and β- (CSN2, csnb) casein genes in reverse orientation on the left and the expansion of the region between β- and kappa- (CSN3, csnk) casein on the right. Except for β-casein, all genes are transcribed from left to right. In monotremes, a recent duplication of CSN2 has led to CSN2b, whereas in eutherians, an ancient duplication produced CSN1S2, which has been duplicated in some species to produce CSN1S2b, now a pseudogene in human but not in mouse. In the marsupial locus, there is no casein duplication and the spacing region contains several copies of an invading repetitive element (black arrows), suggesting active rearrangement of this region in the ancient marsupial lineage, probably resulting in the deletion of a putative ancient casein duplicate in the area. [file gb-2011-12-8-r81-S4.pdf]
